# Spending to grow or growing to spend? Relationship between public health expenditure and income of Indian states

**DOI:** 10.1016/j.ssmph.2022.101310

**Published:** 2022-12-15

**Authors:** Khushboo Balani, Sarthak Gaurav, Arnab Jana

**Affiliations:** a Ashank Desai Centre for Policy Studies, Indian Institute of Technology Bombay, Mumbai, 400076, India; b Shailesh J Mehta School of Management, Indian Institute of Technology, Bombay, India; c Centre for Urban Science and Engineering, Indian Institute of Technology, Bombay, India

**Keywords:** Public health expenditure, Income, Granger causality, Institutions, Inequality

## Abstract

While there are concerns about low public health expenditure in most developing countries, the evidence on the linkage between public health expenditure and income is limited. In countries such as India, where massive public health programmes have been implemented over a period that experienced high economic growth, the relationship between two is a question of interest. We examine the relationship between two at sub-national level, as decisions regarding health are prerogative of the state government. We use data on gross state domestic product and public health expenditure over 1981–2017. Using a robust version of Granger causality that produces reliable results even in presence of parameter instabilities, we find presence of non-linear and bi-directional relationship between the two variables. We report inter-state differences in the income elasticity of health expenditure. These differences can be traced to the differing institutional set up, partly rooted in the administrative decisions taken in colonial India.

## Highlights

• This paper reports bi-directional, non-linear relationship between public health expenditure and income at a sub-national level in India.
• There are differences in the income elasticity of public health expenditure among developed and under-developed states..
• Increased budgetary allocation alone cannot guarantee increased health expenditure by states.

## 1 Introduction

The evidence on the relationship between health expenditure and income has been subject of numerous empirical investigations. However, the focus of most of these studies has largely been on cross-country analysis. The choice of countries in the latter is motivated by availability of data, which meant the sample predominantly consisted of developed countries. The reported results are sensitive to both the timeline chosen (Hartwig, 2010) as well the nature of the causality tests chosen for the analysis. It is no surprise then that the empirical evidence on the relationship between these two variables is mixed, with some studies even reporting no relationship between the two variables, which has implications for the actual budgetary allocation.

The odds are particularly stacked against investments in health and education in developing countries, given the limited availability of financial resources and the long gestation period between investments made and its translation into tangible results. Becker's (2007) analysis of the OECD data succinctly lists the dynamics underlying the linkage between health expenditure and income, and the relationship between the two variables unfolds over many decades. The paper reported the positive impact of increased health spending on income through its linkages to higher life expectancy, higher savings, and investments in education. These arguments, emphasising on different aspects of complementarities between health and education, have been invoked in endogenous growth theory literature as well (Lucas, 1988; Romer, 1990). Increased health expenditure has been linked with better health outcomes (Hall, Swamy, & Tavlas, 2012); increased labour productivity (Raghupathi & Raghupathi, 2020) and higher incomes (Bloom, Canning, & Sevilla, 2004). Yet, to this date, India's public health expenditure as a share of GDP has hovered at around one percent, against the OECD average of eight percent (Organisation for Economic Co-operation and Development, 2019). In an official report on the preparedness for achievement of sustainable development goals for health by 2030, the Comptroller and Auditor General of India observes that the central government's budget allocation for health at INR 650 billion (9 billion USD) for 2019–20 falls way behind the target of one INR 1 trillion rupees (137 billion USD)^1^(GoI, 2019).

While Government of India (Ministry of Health & Family Welfare GoI, 2005) has generally acknowledged that the current levels of expenditure on health are inadequate and there is a need to allocate greater resources, the progress on attaining its own target of 2–3 percent of GDP has largely been dismal in the last two decades. The sub-national analysis becomes important for a vastly populous and geographically diverse country like India. To put things in perspective, the total population of two states namely Uttar Pradesh and Bihar (348 million) is greater than population of USA (331 million), and there are significant differences in the social, cultural and administrative ethos between the northern and southern parts of India, apart from the oft-discussed agro-climatic differences (Paul & Sridhar, 2015). We analyse the relationship between public health expenditure and income at sub-national level as health is classified as ‘state subject’^2^ under the Indian Constitution and the states incur majority of expenditure. This study raises and answers the following questions. First, whether there is a relationship between state-level public expenditure on health and income? Second, what is the nature of this relationship?

In this study, the we focus on 19 states, divided into two groups, based on their demographic characteristics - the eight relatively under-developed, empowered action group (EAG) states and 11 non-EAG states. The EAG states are characterised by fertility levels above the national average and income levels below the national average. The situation is reversed for the non-EAG states. We focus on these two state groups as they constitute the bulk of India's population, and there are structural differences between them with respect to our variables of interest.

The study contributes to an improved understanding of the variations in healthcare spending across states in India. In our study, both state groups have reported presence of a bi-directional relationship between public health expenditure and income in the short-run. This is in contrast to other studies which reported unidirectional relationship between these two variables in the short-run (Behera & Dash, 2018; Pradhan & Bagchi, 2012). We emphasise on the non-linear relationship between two variables as revealed by the robust version of Granger causality developed by Rossi and Wang (2019). The non-linear relationship between the two variables has received limited attention in the literature (Gaies, 2022; Wu, Liu, & Pan, 2014; Ye & Zhang, 2018). Our study, to the best of our knowledge, is first to report non-linearities at the sub-national level in Indian context.

We also highlight the inter-state differences in the nature of the relationship between the two variables across four decades and differences in the income elasticity of public health expenditure. This is despite sizeable transfers, from the national government, to poor-performing states, and the *High-Focus* status accorded to them under National Rural Health Mission (NRHM). We argue that the institutional factors best explain the differences between these two state groups and that health policy's focus on merely increasing budget allocations for the EAG states won't alter their spending trajectory or the quality of utilisation of funds. There is a need to incorporate institutional reform in health policy design, especially in the financial architecture of the state governments, to enhance the effectiveness of public health spending.

This paper is structured as follows. Section 2 presents a brief literature review of the theoretical aspects of the income-health expenditure nexus and some India specific trends. Section 3 describes the data and the methodology employed, Section 4 presents the results, Section 5 presents a discussion of results and Section 6 concludes with key policy implications.

## 2 Review of literature

### 2.1 Review of cross-country evidence

There is vast literature exploring the long-run and the short-run relationship between health expenditure and income for developed countries, but limited number of studies focusing on country-specific dynamics in developing countries (Kouassi, Akinkugbe, Kutlo, & Brou, 2018; Yang, 2020). A few studies have focused on the long-run relationship between healthcare spending and income (Baltagi & Moscone, 2010; Ke, Saksena, & Holly, 2011; Rana, Alam, & Gow, 2020). These studies focus on the time-series analysis of the relationship, whereby understanding co-integration of the series has been an important line of investigation (Hansen & King, 1996; Hitiris & Posnett, 1992; Khan & Ul Husnain, 2019). While the presence of Granger causality between two series is an indication of a short-run relationship, the presence of co-integration confirms presence of a long-run relationship between the given variables. In the context of the empirical relationship between health expenditure and income, three aspects are critical.

First aspect is, whether there is a bi-directional causality (Erdil & Yetkiner, 2009) between healthcare spending and income or a one-way causality (Devlin & Hansen, 2001; Heshmati & Almas, 2001; Ke et al., 2011) or no causality (Hartwig, 2010). The evidence on bi-directional causality is mixed, depending on which income group a country belongs to. In the one-way causality studies, level of GDP has generally been found to be significant, with low and middle-income countries displaying a positive relationship (Halıcı-Tülüce, Doğan, & Dumrul, 2016).

Secondly, in order to attribute causality in the relationship between healthcare spending and income, it is important to acknowledge the endogeneity issues in the estimation. While the endogenous growth theories have made the case for increased health expenditure, due to its linkages with increased human capital formation, the empirical evidence on causal linkage between health expenditure and income has been limited (Anser et al., 2020; Bilgel & Tran, 2013; Sen, 2005). Most studies have restricted themselves to panel data tests of Granger causality, controlling for other factors and cross-sectional dependence. These studies either report evidence in favour of a long-run relationship between the two variables (Baltagi & Moscone, 2010; Gerdtham & Löthgren, 2000; Pradhan & Bagchi, 2012; Wang & Rettenmaier, 2007), or dispute the existence of such relationship (McCoskey & Selden, 1998; Roberts, 2000).

Some of the obstacles in the public health expenditure-income transmission channel are – the substitution effect between public and private expenditure, using the increased public health expenditure for high-cost but low-impact inputs and the inadequacy of complementary infrastructure to access publicly offered services (Bokhari, Gai, & Gottret, 2007). The very nature of budgetary allocation, where spending decisions in current year are a response to poor health outcomes in previous year, also introduces the possibility of endogeneity (Bokhari et al., 2007). In a similar vein, it has been argued that it is harder to change health spending in countries with lower levels of health expenditure growth than in those with higher levels (Tian, Gao, & Yang, 2018), thereby confirming the presence of an inertia in the health expenditure. This has adverse implications for the low-income EAG states, which lag behind the national average on per capita health spending, and given that the poor are affected to a greater extent by variations in health spending (Bidani & Ravallion, 1997).

Thirdly, we also need to emphasise whether the causality we are testing is linear or non-linear in nature. Barring a select few studies (Gaies, 2022; Wu et al., 2014; Ye & Zhang, 2018), the aforementioned studies have focussed on linear causality. This has implications for both empirical evidence and policymaking. The acknowledgement of the non-linearity in the relationship between variables enables to account for the impact of the political processes like regime change and other temporary shocks like business cycle movements. When standard tests reject the absence of causality between health expenditure and income, that evidence is oft cited to argue for the limited impact of health expenditure or increase in health expenditure on income.

### 2.2 India-specific trends

While India has been part of similar cross-country analysis (Arun & Kumar, 2015; Khan & Ul Husnain, 2019; Ye & Zhang, 2018), this study presents disaggregated analysis at the state level, which has received only limited attention in the past (Behera & Dash, 2018; Pradhan & Bagchi, 2012). It was found that state's literacy rate (Rahman, 2008); extent of political participation of the public, implementation of NRHM and per capita income (Hooda, 2015) were the key predictors of the increase in the government health expenditure. The strong explanatory power of state's per capita income implied that central transfers cannot entirely offset the fiscal imbalances across states (Rao & Choudhury, 2012). This meant that the state's own revenue capacity needs to be enhanced. However, in contrast with other developed countries, the horizontal imbalances with respect to revenue capacity have only been increasing in case of India (Rao, 2019).

The state governments incur about 60–70 percent of the total public health expenditure (Central Bureau of Health Intelligence, 2021), but continue to be dependent on Centre for the revenues required for carrying out its functions (Rao & Choudhury, 2008). The situation is particularly adverse for the low-income states, majority of which have relatively low own-tax revenue (Reserve Bank of India, 2020), and are predominantly dependent on the Centre for carrying out its various functions. It was also found that increase in health grants by central government results in substitution of health expenditure by the states from their own resources, with the effect being stronger during times of fiscal distress (Berman, Bhawalkar, & Jha, 2017; Rao & Choudhury, 2012).

## 3 Data and methodology

In context of this study, we focus only on public health expenditure, and have not considered the impact of the out-of-pocket expenditure (OOPE) on health. The relationship between public health expenditure and GSDP is stronger than the relationship between the OOPE on health and GSDP (see Appendix). The implication of these results is that as income increases, state governments are likely to spend more on health than individuals, which is the fundamental policy concern motivating our analysis. The state government's higher propensity to spend than the individuals enable greater internalising of the externalities generated by public health investments, and highlights the regressive nature of the OOPE on health (Wagstaff, Eozenou, & Smitz, 2020).

The data on public health expenditure in India comprises two broad expenditure heads: public health and family welfare. The expenditure on nutrition and sanitation is not incurred by the Ministry of Health and Family Welfare (MoHFW), and is therefore not included in the aggregate public health expenditure. It also excludes the expenditure incurred on health insurance schemes for central government and state government employees (XV Finance Commission, 2021). All the central government grants to a state government, when routed through the state's finance department are reflected in the state government's health budget. However, for a brief period between 2005-06 to 2013–14, there was change in the mechanism of the funding, and some of the central government's funding was directly routed to state health societies. This part of funding, which was not routed through the state government's finance department, is not reflected in the state's budget.^3^

The data on state-wise aggregate public health expenditure (nominal) between 1980 and 1985 is sourced from Duggal, Nandraj, & Shetty (1992). The official public health expenditure data till 1985 included expenditure on water supply and sanitation as well under the health expenditure budget head, and we have utilised a dataset which excludes the water supply and sanitation component, as the expenditure on the latter was not incurred by Ministry of Health and Family Welfare. The public health expenditure data for the remaining period i.e.,1986 to 2017 is sourced from the Economic & Political Weekly Research Foundation (Economic & Political Weekly Research Foundation, 2019). The nominal GSDP time series data for 1981–2011 is sourced from Ministry of Statistics and Programme Implementation (Ministry of Statistics and Program Implementation GoI, 2007; 2008, 2014) and for 2012–17, from Central Statistical Office (Central Statistics Office GoI, 2020)*.*

We test the Granger causality for 19 states over the period 1980–81 to 2016–17 (Budget Estimates). Over this period, state boundaries have been redrawn as new states were formed. In order to have a complete and uniform time series for 37 years, we do not separately incorporate the states of Jharkhand, Telangana, Uttarakhand and Chhattisgarh. Instead, we have two separate categories: the Undivided (where newly formed states' totals are added back to their parent states) and the Divided (which reflects the data of the parent states post bifurcation). The base year used for converting nominal values to real values was 2011–12 which is the current base year for India's retail and wholesale inflation series.

Departing from most studies in the literature, we utilise the non-linear Granger causality test (Rossi & Wang, 2019), instead of utilising the standard test of Granger causality (Granger, 1969). The stationarity of variables is a pre-requisite of the standard test of causality.^4^ In our case, however, both the variables, log GSDP per capita and the public health expenditure were I (1), i.e. not stationary in level form. Using the standard test would amount to changing the null to first differenced series of log real public health expenditure per capita does not Granger cause first differenced series of log real GSDP per capita and vice versa, while the null we are interested in is: log real public health expenditure per capita does not Granger cause log real GSDP per capita and vice versa. To accommodate for the non-stationarity and other parameter instabilities, we utilise the robust version of Granger causality (Rossi & Wang, 2019). There are two other commonly used tests of Non-linear Granger causality (Diks & Panchenko, 2006; Hiemstra & Jones, 1994), but we choose robust version given that it aligns with the characteristics of the dataset.

The robust version of Granger causality explicitly accounts for structural breaks in the data and produces reliable estimates even in presence of parameter instabilities like non-stationarity, absence of co-integration, presence of auto-correlation and heteroscedasticity in the residuals. The robust Granger causality test uses a vector autoregression (VAR) specification, which means that the present values of the log GSDP per capita are regressed against the past values of the log GSDP per capita as well as the past values of log public health expenditure per capita. Besides, the robust version of Granger causality detects the presence of causality, even if it exists for brief time periods.

(1) $$H_{0}:=\varphi_{j,t}^{\omega ,u\;}=0\;\left(standard\;test\right)$$

(2) $$H_{0}:=\varphi_{j,t}^{\omega ,u\;}=0\;\forall_{J}=1,2.\;.\;.n\forall_{t}=1,2\;.\;.\;.\;T\;\left(Robust\;version\;Granger\;Causality\;test\right)$$

(j = cross section unit, t = time, ω&u = variables of interest).

To illustrate this point, the null hypothesis of the standard test of Granger causality (1969), in this case, would be Log of Real Public Health Expenditure per capita (at a given lag) does not Granger cause Log of Real GSDP per capita and vice versa. The null hypothesis of the Robust version of Granger causality would be Log of Real Health expenditure per capita (at a given lag) does not Granger cause Log of Real GSDP per capita for all *time periods*. This last part of the null then facilitates detection of causality, albeit for brief time periods; against the standard test which rejects the null if the causality breaks mid-way. The four test statistics utilised as part of the test are - the exponential Wald test (ExpW), optimal mean Wald test (MeanW), optimal Nyblom test and Quandt likelihood test (SupLR). They take into account the scenarios where alternatives which are proximate (or distant) to (from) the null and the relative constancy (or randomness) of the parameters, and structural breaks (Rossi & Wang, 2019). A detailed examination of the same can be found here (Rossi, 2005). The standard test statistics make fairly strong assumptions about the characteristics of data in terms of presuming a stable growth rate of variables and absence of significant volatility in the data on a yearly basis (Granger, 1969). Given that real world data are less likely to adhere to these assumptions, the aforementioned test statistics were designed to adapt to these changes in the data set, and thereby serve as a robustness check for our analysis. This robust version of Granger causality was run separately for each state given that health is a state subject and regime changes across states were not perfectly synchronous.

The lag selection for Granger causality test were based primarily on the Schwartz-Bayesian Information criterion (SBIC), as t = 37. In cases where the majority of the tests selected a different lag from SBIC, we chose the lag selected by majority. The other tests being Likelihood Ratio test, Akaike Information Criterion, Hannan-Quinn Information criterion and Akaike's Final Prediction Error criterion.

Lastly, we also undertake panel data analysis, by dividing states into two groups, namely EAG and the non-EAG states. This analysis enables to control for unobserved heterogeneity associated with time invariant covariates i.e., unique features of these state groups which influence the strength of the relationship between public health expenditure and GSDP. These time invariant covariates have policy implications as they aid in determining the trajectory of the public health investments in given states. It establishes that the two groups of states behave differently in terms of the responsiveness of the changes in public health expenditure to changes in state's GSDP (both in log per capita terms).

## 4 Results

### 4.1 Granger Causality results

We find bi-directional, non-linear causality between log public health expenditure per capita and log gross state domestic product per capita in the short-run (See Table 1 and Table 2). The term bi-directional causality in this case means that past values of public health expenditure per capita predict present and future values of GSDP per capita and vice versa. With respect to non-linearity, the nature of relationship between these variables in the real world is generally not perfectly linear, as decisions pertaining to budget allocation and economic growth are generally taken in the backdrop of number of political and institutional factors. The standard tests of causality are likely to reject any relationship between these variables because of their assumption about the linear relationship between these two variables. This would mistakenly lead to the implication that these two variables are unrelated, which in turn will impact the allocation of budget for health, especially during the downswings of business cycles (Mooij and Dev, 2002), as it would be erroneously concluded that it does not contribute to growth and recovery. A value of 0, which was present in most cases means that the test results are significant at 0.1 percent level of significance.

**Table 1:** Robust Granger causality results: Health expenditure Granger causes GSDP.

State	ExpW	MeanW	Nyblom	SupLR	Lag
**EAG States**					
Bihar Divided	522.0	351.7	12191.8	1050.4	2
Bihar Undivided	.	392.1	3131.2	1743.4	2
Madhya Pradesh Divided	297.9	117.1	14159.5	602.3	2
Madhya Pradesh Undivided	51.6	43.2	11086.9	109.6	1
Odisha	58.1	33.3	2176.3	122.2	1
Rajasthan	119.0	27.7	14446.4	244.5	1
Uttar Pradesh Divided	158.4	110.0	11851.8	323.2	1
Uttar Pradesh Undivided	132.0	97.0	6512.6	270.5	1
**Non-EAG States**					
Haryana^a^	142.8	42.6	776.0	292.1	1
Kerala	156.1	73.1	14877.2	318.7	1
Karnataka	71.4	29.5	353.3	149.3	1
Maharashtra^a^	59.8	18.5	2577.7	126.1	1
Tamil Nadu	14.0	8.9	1707.9	34.2	1
Andhra Pradesh Undivided	27.2	12.9	5405.7	60.9	1
Himachal Pradesh	91.8	59.7	61918.2	190.1	1
Gujarat	47.5	19.4	7908.9	101.5	1
Jammu & Kashmir	75.2	49.6	16224.9	156.9	1
Punjab	39.6	25.1	8402.1	85.5	1
West Bengal	136.9	50.2	7235.2	280.3	1

a.Note: The results for all the four test-statistics were found to be significant at 1% level.

b Also, the state-wise public health expenditure data for the years 2005–06 to 2013–14 is an underestimation, because it does not include the part of Centre's contribution to the state budget as part of the NRHM. The Centre transferred the funds as part of the scheme directly to the State Health Societies and only the part which was contributed by the States was reflected in the State's budget.

a For states of Haryana and Maharashtra, instead of choosing the lag order based on the one the selected by majority of criterion, i.e. three and four respectively, we choose the lag order as one based on HQIC and SBIC, as the matrix in STATA 16 generated missing values for a higher lag order.

**Table 2:** Robust Granger causality results: GSDP Granger causes Health expenditure.

State	ExpW	MeanW	Nyblom	SupLR	Lag
**EAG States**					
Bihar Divided	.	699.1	184541.3	11627.9	2
Bihar Undivided	.	349.9	317204.9	4452.9	2
Madhya Pradesh Divided	80.5	51.3	27762.5	167.5	2
Madhya Pradesh Undivided	58.5	20.9	5558.3	123.4	1
Odisha	58.0	65.9	31228.7	122.5	1
Rajasthan	205.8	101.9	25292.6	418.1	1
Uttar Pradesh Divided	172.5	89.5	16187.6	351.4	1
Uttar Pradesh Undivided	76.2	76.6	17576.1	158.1	1
**Non-EAG States**					
Haryana^a^	51.6	23.8	27862.6	109.6	1
Kerala	77.8	45.9	40827.9	162.1	1
Karnataka	225.8	92.5	18564.6	458.0	1
Maharashtra^a^	108.6	95.9	19320.1	223.7	1
Tamil Nadu	71.2	45.7	44278.7	148.9	1
Andhra Pradesh Undivided	40.3	23.3	19101.3	87.1	1
Himachal Pradesh	88.3	64.5	86855.1	183.1	1
Gujarat	350.5	81.9	23195.9	707.5	1
Jammu & Kashmir	107.3	129.7	259271.9	221.0	1
Punjab	199.2	58.8	17977.1	404.9	1
West Bengal	370.4	166.5	33610.9	747.1	1

a Note: The results for all the four test-statistics were found to be significant at 1% level.

b Also, the state-wise public health expenditure data for the years 2005–06 to 2013–14 is an underestimation, because it does not include the part of Centre's contribution to the state budget as part of the NRHM. The Centre transferred the funds as part of the scheme directly to the State Health Societies and only the part which was contributed by the States was reflected in the State's budget.

a For states of Haryana and Maharashtra, instead of choosing the lag order based on the one the selected by majority of criterion, i.e. three and four respectively, we choose the lag order as one based on HQIC and SBIC, as the matrix in STATA 16 generated missing values for a higher lag order.

Our results are in contrast to Behera and Dash (2018) who found bi-directional relationship between the two variables in the long-run, while a unidirectional relationship running from GSDP to public health expenditure in the short-run. Pradhan and Bagchi (2012) reported bi-directional relationship only for states of Bihar, Madhya Pradesh, Meghalaya, West Bengal and India as a whole. Both these studies utilised the standard test of Granger causality. Our study, to our knowledge, is the first one to test non-linear causality between public health expenditure and GSDP at a sub-national level in India. The Ye and Zhang (2018) study, which utilises the Diks and Panchenko test of non-linear Granger Causality, reports absence of non-linear causality for India as whole in their cross-country analysis.

While the causality running from GSDP to health expenditure was found less likely to break for states, the causality running from health expenditure to GSDP does break down at multiple points for states (See Web Appendix).^5^ These are important findings for of the following reasons. Firstly, breakdown of the causality implies that income alone cannot be a predictor of increased public health expenditure, thereby implying changing political regimes and administrative capacity of the state governments have an impact on this causality as well. Thus, economic growth is only a necessary, but not a sufficient condition to ensure increased health expenditure and health outcomes (Hooda, 2013; Rahman, 2008). For instance, factors like political competitiveness (Datta, 2020), two party versus multi-party competition (Chhibber & Nooruddin, 2004), party continuation^6^ (Youkta & Paramanik, 2020) have found to have a positive impact on health spending in states in India. Second, the presence of causality running from health expenditure to GSDP, builds an economic case for increase in public health expenditure, beyond the traditional arguments in context of social welfare.

The causality running from per capita GSDP to per capita public health expenditure does not break for majority of states, even once, in the 37 years. This implies that on average, past values of real per capita GSDP are a good predictor of future values of real per capita public health expenditure. For states of Undivided Andhra Pradesh, Kerala, Tamil Nadu, Haryana, Divided Madhya Pradesh and Undivided Madhya Pradesh, the relationship between the two variables breaks down for select few years in between. In case of Undivided Andhra Pradesh, Tamil Nadu, Haryana, Divided Madhya Pradesh, Undivided Madhya Pradesh, the rate of change in the real per capita public health expenditure has largely been stable, while the yearly change in real GSDP per capita has seen greater volatility, especially post early 2000s.

The breaks in case of Undivided Andhra Pradesh can largely be explained in terms of health being an important priority for the ruling party across election and business cycles, which ensured the relative stability in the budget allocation towards health. Incidentally, the state also has benefitted from relative stability in civil service posted in the Ministry of Health and Family Welfare, where the average tenure of the IAS officer is 557 days, the highest among the major states, as per the dashboard created by How India Lives. Even in case of Tamil Nadu, the institutionalisation of health reforms meant that the regime-change effect was negligible and the health expenditure did not respond to changes in gross state domestic product.

In case of Haryana, the breakdown corresponds with regime change and there is break in causality for almost a decade (2000–10). The break in causality observed in both Kerala and Haryana post 2005, can be partly attributed to the introduction of NRHM which was a centrally sponsored scheme. The impact of the latter on the state's spending in the initial years could not be completely captured because funds were being devolved through the state health societies, and so were not reflected in state government's health budget. In case of Divided Madhya Pradesh, the break in causality is caused due to the partition in 2001, because there is a sharp fall in GSDP. In case of Undivided Madhya Pradesh, there is break in causality for almost 20 years, from 1994 to 2004; there have been relatively greater fluctuations in GSDP of Madhya Pradesh in comparison to other states, since beginning of study period.

In context of second part of this causality, whether past values of public health expenditure are a good predictor of future values of GSDP per capita, the evidence is mixed. In case of Jammu & Kashmir, Kerala, Undivided Bihar, Divided Bihar, Undivided Madhya Pradesh, Divided Madhya Pradesh, Divided Uttar Pradesh and Undivided Uttar Pradesh, the causality does not break even once, across four decades. Since this list comprises six of the eight EAG states, it underscores the importance for continued public investment in healthcare in improving the economic condition of these states. The EAG states have had lowest levels of per capita public expenditure levels, and this evidence makes an economic case for the increased public health expenditure. This is an important result because the evidence on positive economic implications resulting from increased health expenditure has generally been limited, and increase in health expenditure is generally argued because of its social implications (Bloom et al., 2004).

In the case of Odisha, the causality breaks at multiple points, at least once in every five years, but is present since 2006. This can be partly attributed to the presence of a stable regime over the last 20 years and the greater focus of the state on the public health reforms in the recent times. In case of Rajasthan, the causality is largely absent across all the years, except for select five years (2000–05). In case of non-EAG states, majority of them experience a breakdown in causality. The states of Tamil Nadu, Haryana, Maharashtra, Gujarat, Undivided Andhra Pradesh are the ones where there is existence of causality for only very brief periods of time. The poor predictability of public health expenditure in determining the trajectory of GSDP has largely to do with the presence of high-income inequality in the developed states (Gradin, 2018). The differential impact of health spending largely stems from differing levels of need and ability to substitute private spending for public spending for people from different socio-economic strata (Farahani, Subramanian, & Canning, 2010).

### 4.2 Panel data analysis

We also present panel data analysis, to further highlight the differences in responsiveness of the EAG states (Divided and Undivided) and non-EAG states to changes in GSDP. The better performing states, i.e., non-EAG states, report lower elasticity in public health expenditure per capita with respect to changes in per capita GSDP, which also points to the relatively greater availability of fiscal space, even during economic downturns. The log GSDP per capita co-efficient was found to be less than 1 in our panel data analysis (see equation (3) and Table 3), reiterating the necessity characteristic of the public health expenditure. In another study of 16 major states, between 1988 and 2012, the reported co-efficient of state's income for EAG states was about 0.67, while it was 0.41 in case of non-EAG states (Hooda, 2015).

**Table 3:** Panel data analysis.

	(1)	(2)	(3)	(4)	(5)	(6)
lnHPc	lnHPc	lnHPc	lnHPc	lnHPc	lnHPc	lnHPC
lnGSDPpc	0.804*** (0.0317)	0.792*** (0.0781)	0.487*** (0.0340)	0.592*** (0.0249)	0.804*** (0.0318)	0.771*** (0.0750)
_cons	−2.621*** (0.321)	−2.503* (0.790)	0.845* (0.365)	−0.282 (0.267)	−2.620*** (0.322)	−2.290* (0.757)
**N**	185	185	407	407	185	185
**Fixed Effects**	No	Yes	No	Yes	No	Yes
**Divided**	No	No	NA	NA	Yes	Yes
**EAG**	Yes	Yes	No	No	Yes	Yes

* p < 0.05, ** p < 0.01, *** p < 0.001.

a Standard errors in parentheses.

b Note: We have used only Balanced Panels for our analysis. No in Fixed Effects row indicates Base OLS regression results, No in Divided Row means it is Undivided States Panel, No in EAG row means it is non-EAG states panel.

**Fig. 3:**
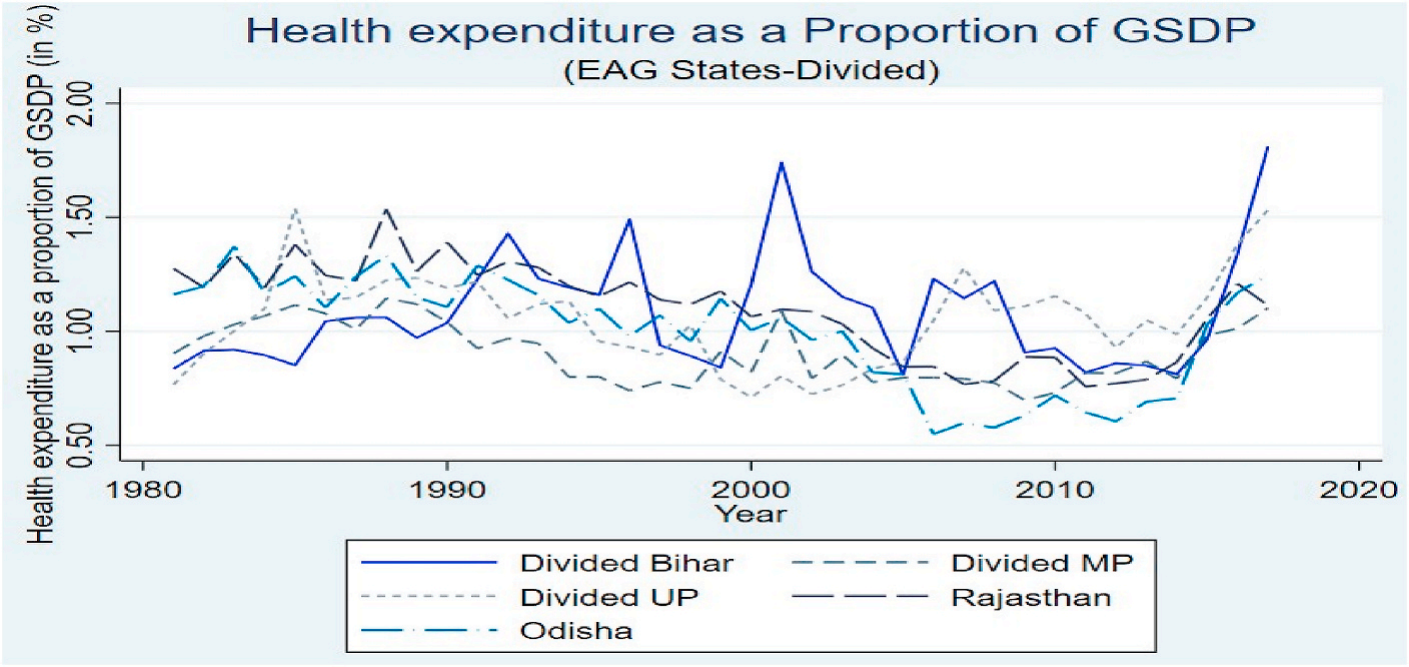
Expenditure on health as a proportion of GSDP (EAG states-Divided). **Source**: Data on state-wise aggregate public health expenditure (nominal) between 1980 and 1985 is sourced from (Duggal et al., 1992). The data for the remaining period i.e.,1986 to 2017 is sourced from the Economic & Political Weekly Research Foundation (Economic & Political Weekly Research Foundation, 2019). Nominal GSDP time series data for 1981–2011 is sourced from Ministry of Statistics and Programme Implementation (Ministry of Statistics and Program Implementation GoI, 2007; 2008, 2014) and for 2012–17, from Central Statistical Office (Central Statistics Office GoI, 2020). *Estimates for 2017 are Budget Estimates #States of Chhattisgarh, Jharkhand and Uttarakhand were formed in 2001 from reorganisation of states of Madhya Pradesh, Bihar and Uttar Pradesh, respectively.

**Fig. 4:**
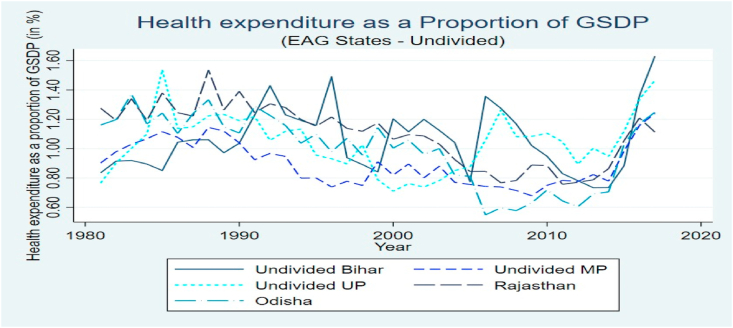
Expenditure on health as a proportion of GSDP (EAG states-Undivided). **Source**: Data on state-wise aggregate public health expenditure (nominal) between 1980 and 85 is sourced from (Duggal et al., 1992). The data for the remaining period i.e.,1986 to 2017 is sourced from the Economic & Political Weekly Research Foundation (Economic & Political Weekly Research Foundation, 2019). Nominal GSDP time series data for 1981–2011 is sourced from Ministry of Statistics and Programme Implementation (Ministry of Statistics and Program Implementation GoI, 2007; 2008, 2014) and for 2012–17, from Central Statistical Office (Central Statistics Office GoI, 2020). *Estimates for 2017 are Budget Estimates #States of Chhattisgarh, Jharkhand and Uttarakhand were formed in 2001 from reorganisation of states of Madhya Pradesh, Bihar and Uttar Pradesh, respectively.

**Fig. 5:**
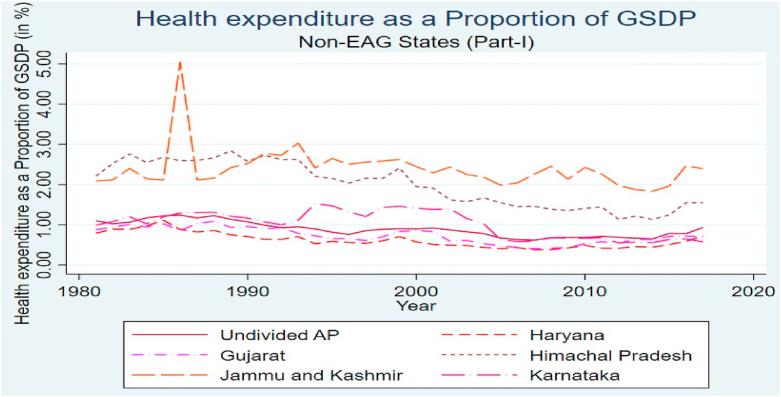
Expenditure on health as a proportion of GSDP (Non-EAG states-Part I). **Source**: Data on state-wise aggregate public health expenditure (nominal) between 1980 and 85 is sourced from (Duggal et al., 1992). The data for the remaining period i.e.,1986 to 2017 is sourced from the Economic & Political Weekly Research Foundation (Economic & Political Weekly Research Foundation, 2019). Nominal GSDP time series data for 1981–2011 is sourced from Ministry of Statistics and Programme Implementation (Ministry of Statistics and Program Implementation G. of I, 2007; 2008, 2014) and for 2012–17, from Central Statistical Office (Central Statistics Office G. of I, 2020). *Estimates for 2017 are Budget Estimates

**Fig. 6:**
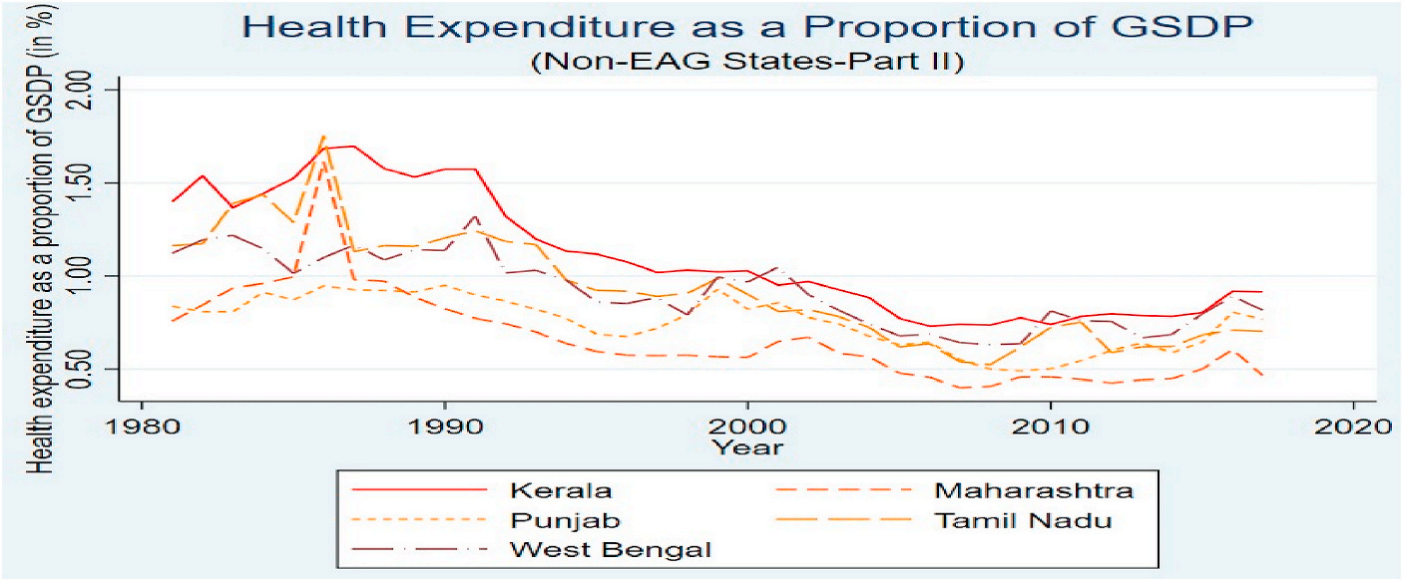
Expenditure on health as a proportion of GSDP (Non-EAG states-Part II). **Source**: Data on state-wise aggregate public health expenditure (nominal) between 1980 and 85 is sourced from (Duggal et al., 1992). The data for the remaining period i.e.,1986 to 2017 is sourced from the Economic & Political Weekly Research Foundation (Economic & Political Weekly Research Foundation, 2019). Nominal GSDP time series data for 1981–2011 is sourced from Ministry of Statistics and Programme Implementation (Ministry of Statistics and Program Implementation G. of I, 2007; 2008, 2014) and for 2012–17, from Central Statistical Office (Central Statistics Office G. of I, 2020). *Estimates for 2017 are Budget Estimates.

Similar differences between the EAG and non-EAG states, in terms of the income co-efficient, emerged in our analysis as well, 0.79 (EAG-Undivided), 0.77 (EAG-Divided) versus 0.59 (non-EAG). The smaller co-efficient of the non-EAG states implies that public expenditure of these states is less responsive to changes in income (relatively income inelastic) compared to the EAG states. Interestingly, the non-EAG states have spent lower amounts as a percentage of GSDP than EAG states, but it is the relative year-on-year consistency in the spending which appears to have paid off for them in terms of better health outcomes (see Figs. 3–6 in Appendix).

(3) $$\ln\;\left({PercapitaHealthexp}_{it}\right)=\beta_{0\;}+\beta_{1}\;\ln\left(Percapita{GSDP}_{it}\right)+\alpha_{i}+\varepsilon_{it}$$

The differences in the co-efficient between the undivided and divided panel in the EAG states also points to differences emerging in spatially contiguous and culturally similar, but now administratively different regions. This phenomenon warrants deeper examination. These differences arising from spatial units have not received enough attention despite the fact that the administrative units may acquire a unique identity either by design or through practice, without any corresponding basis in ethnicity, race or religion (Kanbur, 2006). The spatial inequality of this nature then underscores the salience of institutional divergence (Acemoglu & Robinson, 2012) that explains the conundrum of inequalities in the spending and health outcomes in geographically contiguous, but administratively different regions. The newly formed states in 2001, namely Jharkhand, Uttarakhand and Chhattisgarh have indeed traced differing trajectory of public health spending compared to their parent states, often with mixed results with respect to health outcomes (Jose, 2019).

## 5 Discussion

The above results point to the existence of a bi-directional relationship between public health expenditure and income, albeit in the backdrop of differing dynamics across two state groups. We argue that these differences cannot be corrected by fiscal reforms alone or by merely increasing budgetary allocation to alleviate inter-state inequalities in public health spending. As we observed in our results, that the breaks in causality between public health expenditure and income coincided with regime change, and not with business cycle movements, which have implications for budgetary constraints. We argue that the inter-state differences in health spending and income largely stem from differences in the institutional arrangements,^7^ which in turn also had implications for the extent of presence of the private health sector (Duggal, 2020), quality of governance and state capability. This is in line with the argument that the cross-sectional differences are a result of differences in institutions, as technological diffusion largely happens at the same rate at a point in time (Deaton, 2013; Filmer & Pritchett, 1999; Weil, 2014). It was with respect to complementary reforms, alongside the introduction of new technology, where the gap between the progress of developing and developed countries was more apparent (Weil, 2014).

Both technology diffusion and increase in funding are attractive pursuits from populist perspective, as they are relatively easily actionable measures and the resultant output is apparent in a relatively short span of time. Building state capability and institutional capacity, on the other hand is a relatively complex, less actionable and a more long-drawn exercise (Andrews, Pritchett, & Woolcock, 2017), which necessitate a long-term commitment across different regimes. The 15th Finance Commission (2021–2026) report also acknowledged that the apparent causes for lagging performance of the public health in the country are a reflection of governance issues at the lowest levels. And these governance issues faced by weakest states in India cannot be resolved by mere implementation of the technical solutions outlined by health ministry (Peters, Rao, & Fryatt, 2003).

One of the events in history which is believed to be a starting point of these differences is the differing land revenue collection system introduced by the British in different regions of India (Banerjee & Iyer, 2005). They explain the differences in the economic trajectory of the states in terms of whether or not there was a pre-dominance of *zamindari* system (land rights were vested with the landlord as against cultivators) in colonial India. This system was implemented only in Bengal, Bihar and Odisha, and in select pockets in Southern India. The *zamindari* system's emphasis on collection of land revenue at pre-determined rates, and without any link with the actual agricultural produce resulted in lack of any incentive on part of the farmers or landlords to improve the productivity of the land. It was largely the present-day EAG states which received lowest share of the land revenue collected by them under this system (Mukherji & Mukherji, 2012). For instance, in 1887, the Bombay Presidency received 60 percent of all its land revenue, while Bengal Presidency (consisting of present-day West Bengal, Bihar, and Odisha as well Bangladesh till partition in 1905) received only 32 percent of its total revenue (Ibid).

This system resulted in lower public development expenditures (Banerjee & Iyer, 2005), and lower administrative effort (Mukherji & Mukherji, 2012) vis-à-vis regions where land rights remained with cultivators. Citing the Memorandum for the Indian Statutory Commission on the Working of the reforms in Bihar and Odisha (1930), Ghosh (2007) highlights the vast differences in administrative capacity, with there being one police officer per 776 people in Bombay Presidency versus one for every 2,372 people in Bihar and Odisha. Thus, the differences in administrative, health and education expenditure had already started to emerge in late 19th century and nearly 150 years later (see Table 4), they have continued to persist. A similar path dependence explanation was also put forth in explaining the poor performance of the EAG states in the post-independence period, which despite their substantial natural resource endowment, have lagged behind on income and health indicators (Corbridge, 2010).

**Table 4:** Expenditure on general administration and health in colonial India (per 1000.).

Province	General Administration		Health	
	1867–77	1927–28	1876–77	1927–28
Bombay	374	411	285	141
Central	185	169	142	53
Madras	159	193	139	98
Punjab	244	190	135	126
United	140	103	78	51
Bengal	100	100	100	100
Assam	159	136	82	121
Bihar and Odisha	NA	75	NA	51

a. Note: Central, Bengal and United Provinces map with the present day EAG states. Bihar and Odisha were split from the Bengal Presidency in 1912. The expenditure on Bengal is assumed to be 100 and the expenditure of the remaining is a ratio of that.

The existence of governance issues and rampant corruption in the EAG states has adversely impacted the utilisation of the funds.^8^ The utilisation ratio of NHM funds in some of the EAG states was less than 50 percent for 2016–17 – Bihar (44%), Jharkhand (48%) and Uttar Pradesh (45%) (Choudhury & Mohanty, 2019), which defeats the whole purpose of National Health Mission propelling the expenditure of the High-Focus states. The low utilisation impacts the state's ability to garner funds in subsequent years, as it is partly contingent on producing the requisite utilisation certificates of the previous year (Venkateswaran, 2021). It is also important to note that the utilisation was poor in components which entailed greater complexity and sometimes necessitated innovation, like procurement of drugs, undertaking capital expenditure and other strategic decisions (Choudhury & Mohanty, 2020). It has been argued that the sustained low public investments have resulted in higher dependence on private sector, thereby resulting in higher OOP burden in these states (Duggal, 2020).

## 6 Conclusion and policy implications

In this paper we tested the presence of non-linear Granger causality between public health expenditure and income at a sub-national level in India. The presence of Granger causality indicates a short-run relationship between the two variables however, the term short-run is used here in technical terms and not to refer to actual time period. The relationship between these two variables has received only limited attention in the Indian context, and studies have generally reported a unidirectional and linear relationship between the two variables. This is the first attempt to comprehensively study this relationship at a sub-national level, with a special focus on the EAG and the non-EAG axis. While there is a bi-directional relationship between these two variables, the role of institutional factors and governance is equally important in ensuring that increase in one of these variables necessarily translates into increase in the other. We argue that higher spending on health and higher budgetary allocations alone will not contribute to better health outcomes and higher income as these variables do not operate in a vacuum, but are rooted in the political economy of the given states. This is borne out by cross-country experience as well, where despite having highest per capita public health expenditure, USA's health outcomes and income performance lags behind its OECD peers (Wager, Ortaliza, & Cox, 2022; World Bank & OECD National Accounts, 2022).

The institutional differences argument lends support to the need for specific policy focus on administrative and governance reforms in the EAG states. The NHM's focus on increasing allocation is only a partial solution, as increased allocation has not translated into increased expenditure for these states. It has been found that if the entire allocated sum was utilised NHM funds had the potential to reduce the inter-state co-efficient of variation in per capita public health spending from 0.82 to 0.67, but in reality it could reduce it to only 0.71 (Choudhury & Mohanty, 2020). Thus, the differing patterns of expenditure across state groups necessitate greater need for bottom-up approach to health policy planning. At the central level, there is a need to move away from the current input-based budgeting towards output-based, to further incentivise the improvements in the utilisation of funds.

In this paper, we argue that increased public health expenditure contributes to increase in GSDP across states of India. We acknowledge that the Granger causality test does not establish the causality in true sense of the term, rather it is used for predictive causality that is to determine whether one time series is useful in forecasting another (Devlin & Hansen, 2001; Diebold, 2007, pp. 230–231). The instrumental regression framework is not utilised to establish the causality, due to lack of availability of reliable instruments for both variables for the given time periods. The emphasis on establishing strict exogeneity as part of the method has made finding good instruments more of an exception, than norm (Deaton, 2019). We rely on theoretical motivation and the acknowledgement by the national government that the health sector is in need of reform and greater public investment (XV Finance Commission, 2021).

We also emphasise the non-linearity of the relationship between health expenditure and income, the dynamics of which have not been adequately studied at both national and international level. This is an important line of enquiry, especially understanding the role of macroeconomic shocks, demographic factors, technology and political factors in mediating the relationship between two variables. It will also answer, at the more granular level, questions pertaining to the nature of returns to health expenditure and the presence of thresholds for regions with given characteristics, beyond which the returns to increase in per capita public health expenditure are diminishing.

## Ethical statement

1) This material is the authors' own original work, which has not been previously published elsewhere.
2) The paper is not currently being considered for publication elsewhere.
3) The paper reflects the authors' own research and analysis in a truthful and complete manner.
4) The paper properly credits the meaningful contributions of co-authors and co-researchers.
5) The results are appropriately placed in the context of prior and existing research.
6) All sources used are properly disclosed (correct citation).
7) All authors have been personally and actively involved in substantial work leading to the paper, and will take public responsibility for its content.

## Authorship statement

All persons who meet authorship criteria are listed as authors, and all authors certify that they have participated sufficiently in the work to take public responsibility for the content, including participation in the concept, design, analysis, writing, or revision of the manuscript. Furthermore, each author certifies that this material or similar material has not been and will not be submitted to or published in any other publication before its appearance in the *Social Science and Medicine – Population Health*.

Category 1: Conception and design of study: K.B. Balani, S.G. Gaurav, A.J. Jana; acquisition of data: K.B. Balani, S.G. Gaurav; analysis and/or interpretation of data: K.B. Balani, S.G. Gaurav, A.J. Jana.

Category 2: Drafting the manuscript: K.B. Balani; revising the manuscript critically for important intellectual content: K.B. Balani, S.G. Gaurav, A.J. Jana.

Category 3: Approval of the version of the manuscript to be published (the names of all authors must be listed): Khushboo Balani, Sarthak Gaurav, Arnab Jana.

## Declaration ethics approval and consent to participate

Not Applicable.

## Consent for publication

Not Applicable.

## Availability of data and materials

While majority of data used in the analysis is available in public domain, as mentioned in the main text, the adjusted health expenditure datasets used during the current study are available from the corresponding author on reasonable request.

## Funding

None.

## Declaration of competing interest

The authors declare that they have no competing interests.

## Figures and Tables

**Fig. 1:**
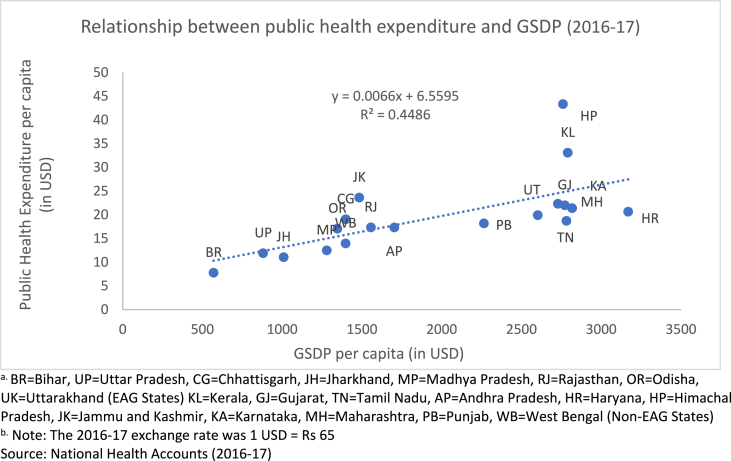
Relationship between public health expenditure per capita and GSDP per capita. a. BR=Bihar, UP=Uttar Pradesh, CG=Chhattisgarh, JH = Jharkhand, MP = Madhya Pradesh, RJ = Rajasthan, OR=Odisha, UK=Uttarakhand (EAG States) KL=Kerala, GJ = Gujarat, TN = Tamil Nadu, AP = Andhra Pradesh, HR=Haryana, HP=Himachal Pradesh, JK = Jammu and Kashmir, KA=Karnataka, MH = Maharashtra, PB=Punjab, WB=West Bengal (Non-EAG States). b. Note: The 2016–17 exchange rate was 1 USD = Rs 65. Source: National Health Accounts (2016–17)

**Fig. 2:**
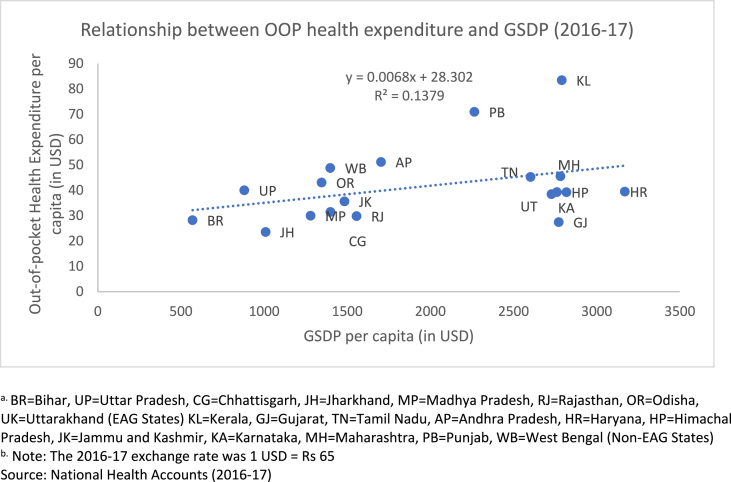
Relationship between OOP payments per capita and GSDP per capita. a. BR=Bihar, UP=Uttar Pradesh, CG=Chhattisgarh, JH = Jharkhand, MP = Madhya Pradesh, RJ = Rajasthan, OR=Odisha, UK=Uttarakhand (EAG States) KL=Kerala, GJ = Gujarat, TN = Tamil Nadu, AP = Andhra Pradesh, HR=Haryana, HP=Himachal Pradesh, JK = Jammu and Kashmir, KA=Karnataka, MH = Maharashtra, PB=Punjab, WB=West Bengal (Non-EAG States). b. Note: The 2016–17 exchange rate was 1 USD = Rs 65. Source: National Health Accounts (2016–17)

## Data Availability

Data will be made available on request.

## Appendix

### Link for Web Appendix

https://drive.google.com/file/d/13NuOVwNPDuxtw8SWvSpGfsBcTrJvWm8y/view?usp=sharing.

## Footnotes

1 The exchange rate used here is 1 USD = 73 INR.

2 The state subject constitutes the list of items where the respective state government has the complete authority to take decisions on the funding and functioning of the listed items.

3 There is no single, comprehensive, and publicly available source to track the funding that state health societies received directly from the central government during this time period.

4 The more appropriate term for Granger causality would have been Granger predictability (Stock & Watson, 2012).

5 The blue line in the figures in Web Appendix traces the presence of causality between the two variables. For all the time periods, where the blue line lies above the 5% and 10% threshold, it implies presence of causality, and the for the time periods where it is below those thresholds, it implies absence of causality.

6 The impact of party continuation was significant only for select states, largely the non-EAG group and select north-eastern states.

7 We also rule out the differences in physical environment as an explanation for the inter-state health expenditure and income inequalities in India. We observe that the tropical and sub-tropical zones overlap across the EAG-non-EAG states, and the differences amongst the EAG states post bifurcation point to the role of institutions in impacting health expenditure as well as income.

8 The financial architecture of the scheme in fact under-states the extent of under-utilisation. A large sum of unspent balances is with the implementing agencies, namely the State Health Societies, after being released by Central and State budgets, so they were in fact recorded as expenditures in government budgets! (Choudhury & Mohanty, 2020).
