# Supplementary material for: Spending to grow or growing to spend? Relationship between public health expenditure and income of Indian states
Source: SSM Popul Health. 2022 Dec 15;21:101310. doi: 10.1016/j.ssmph.2022.101310 (PMC9795522; doi:10.1016/j.ssmph.2022.101310)
Supplement: Multimedia component 1 [file mmc1.pdf]

Figure 1

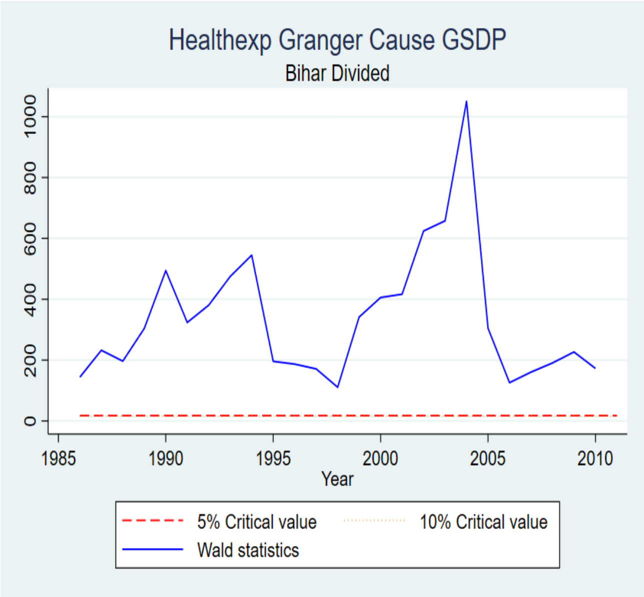

Figure 2

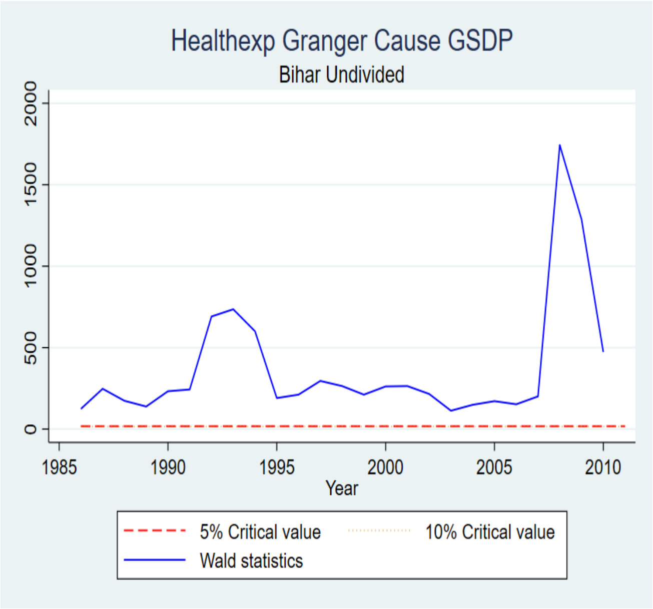

Figure 3

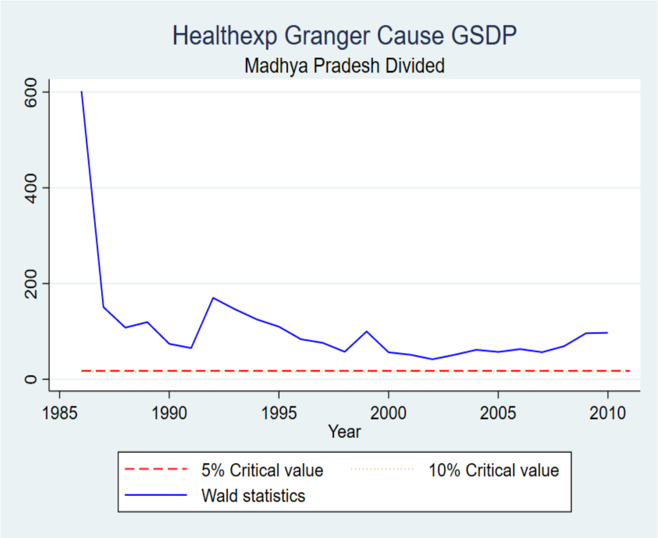

Figure 4

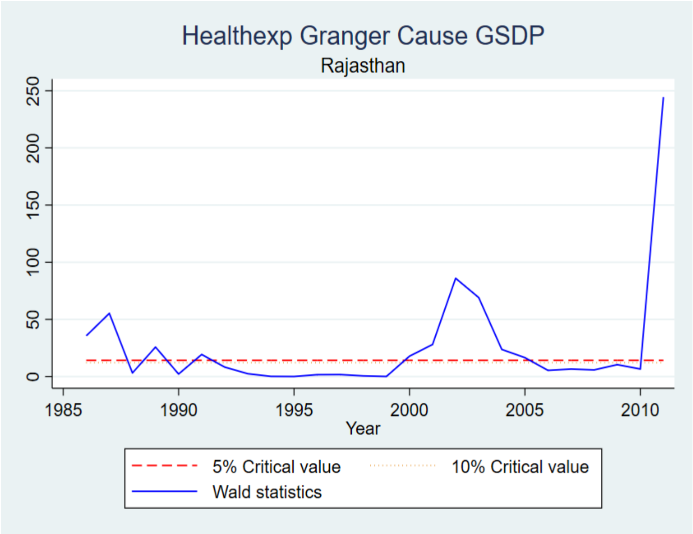

Figure 5

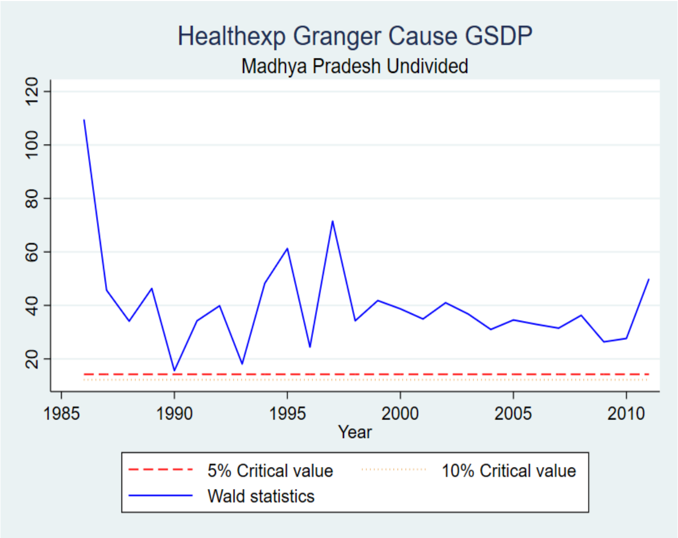

Figure 6

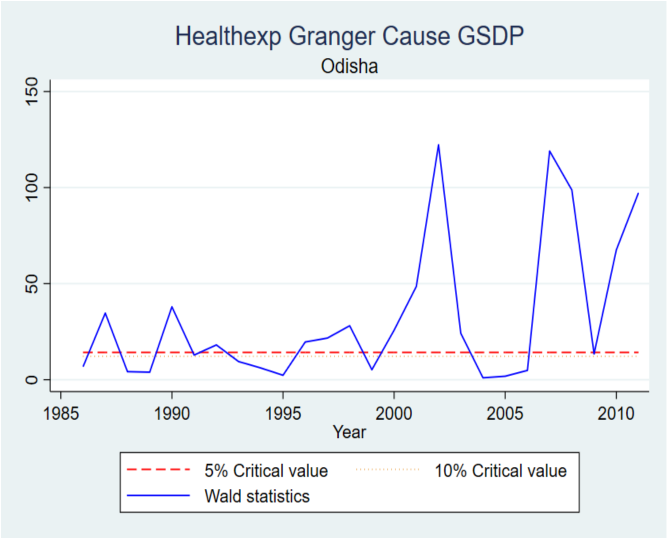

Figure 7

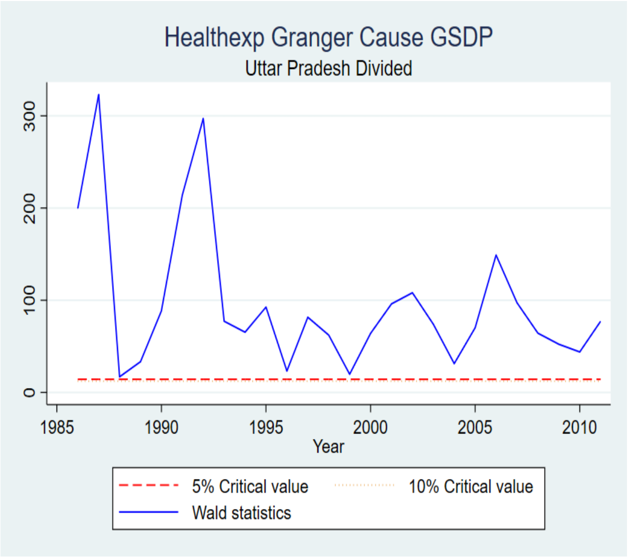

Figure 8

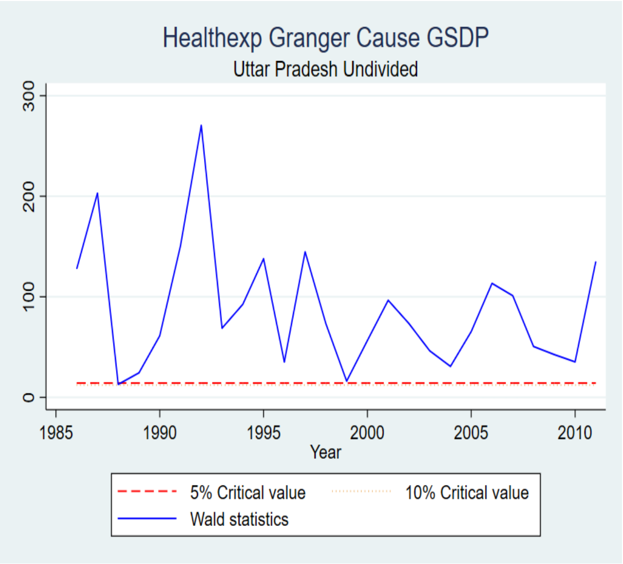

Figure 9

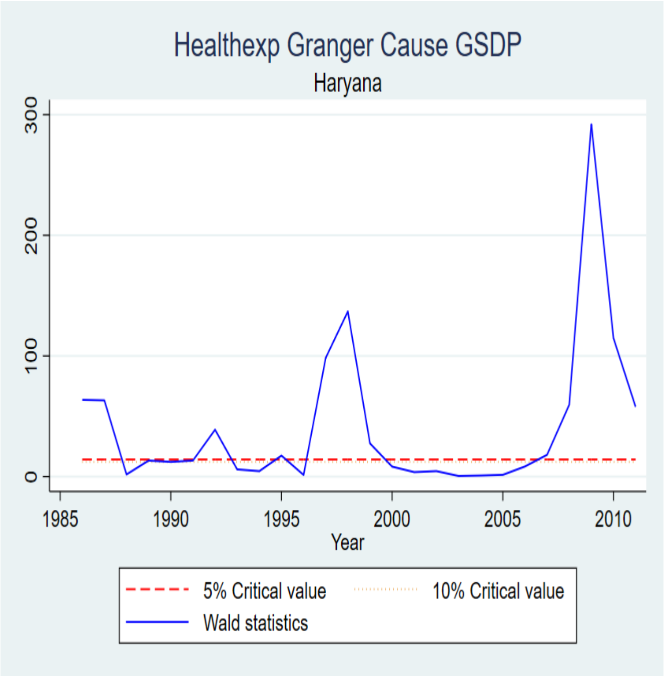

Figure 10

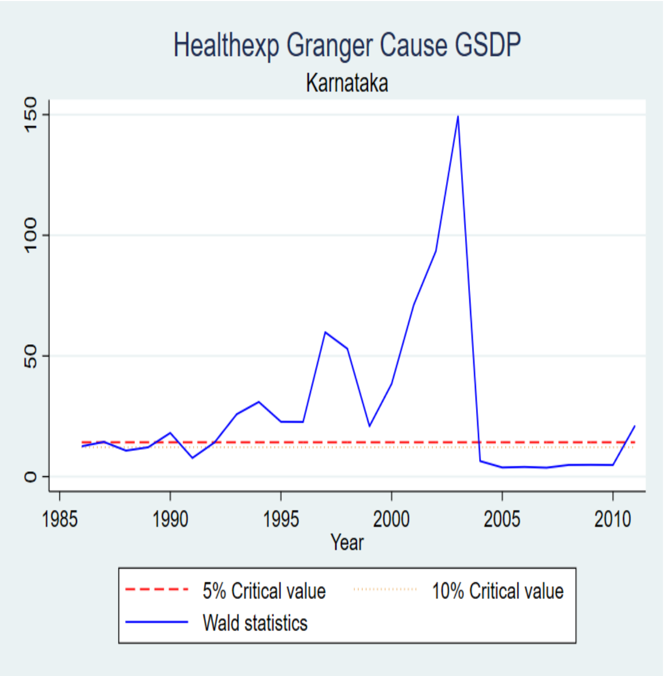

Figure 11

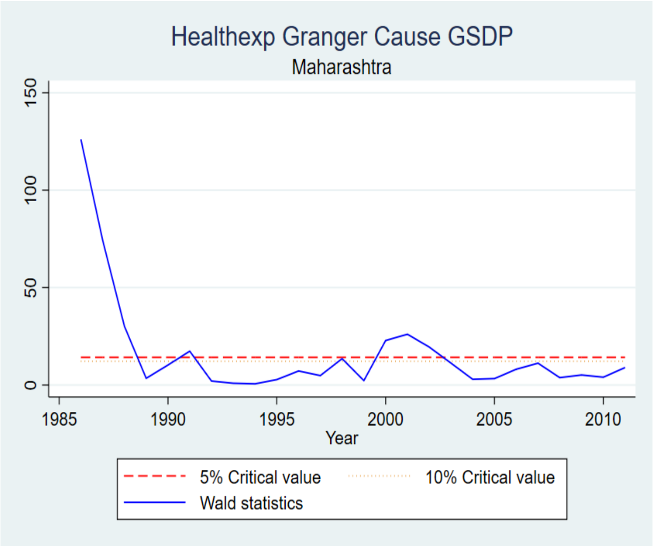

Figure 12

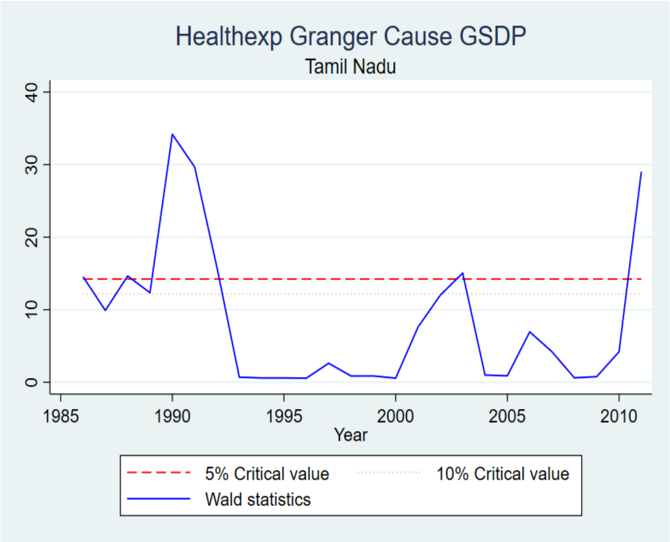

Figure 13

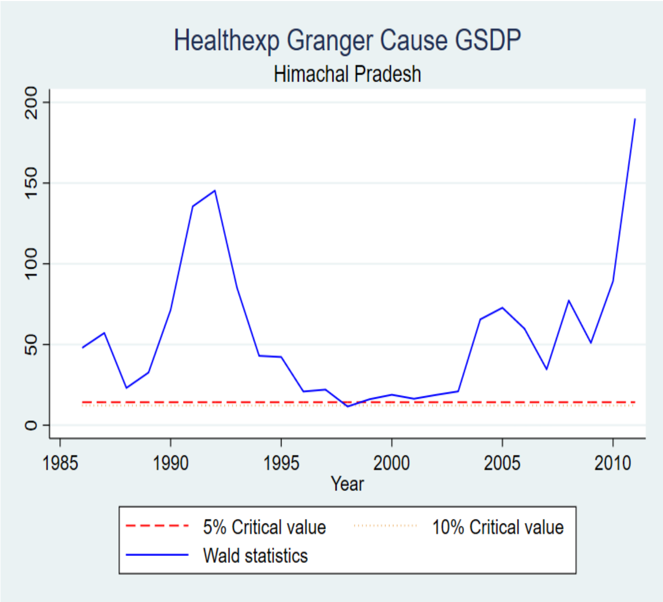

Figure 14

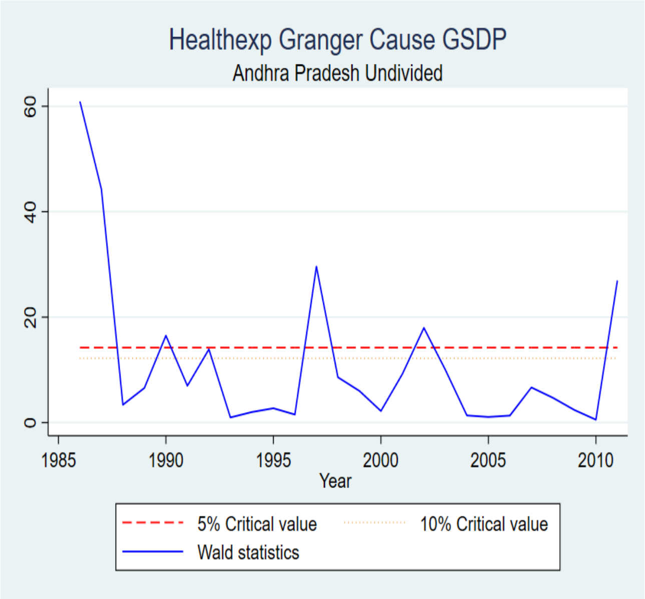

Figure 15

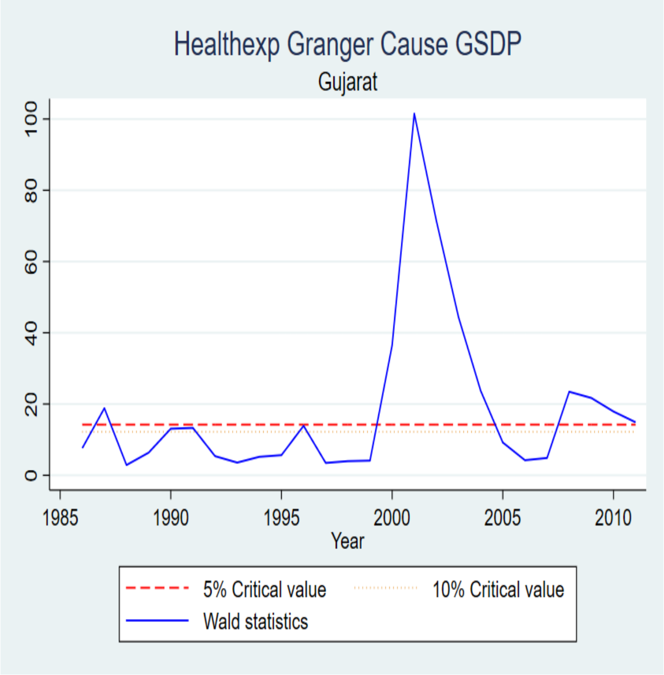

Figure 16

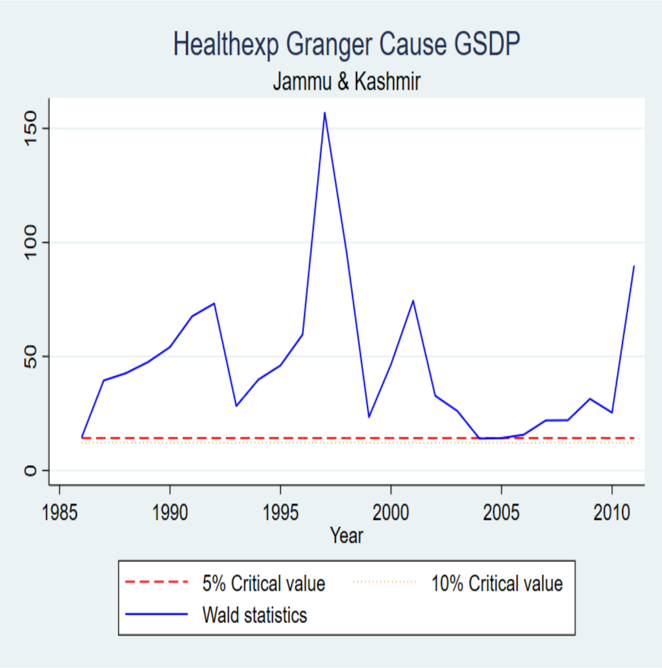

Figure 17

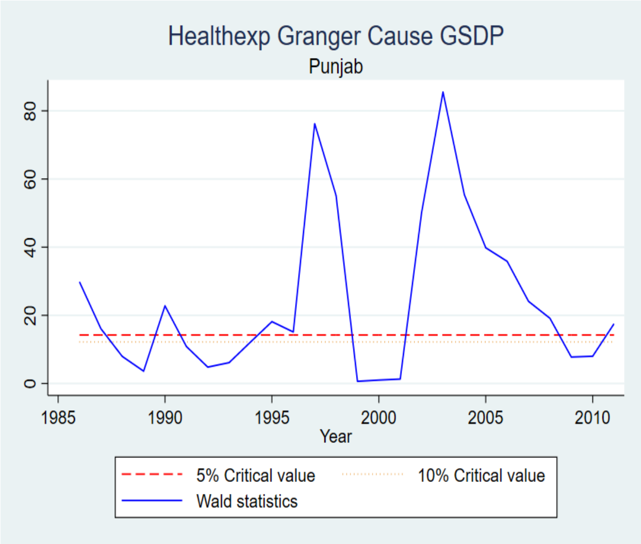

Figure 18

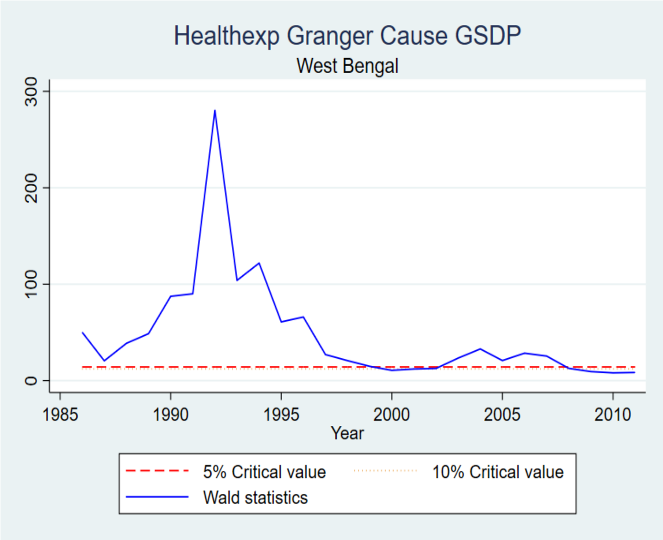

Figure 19

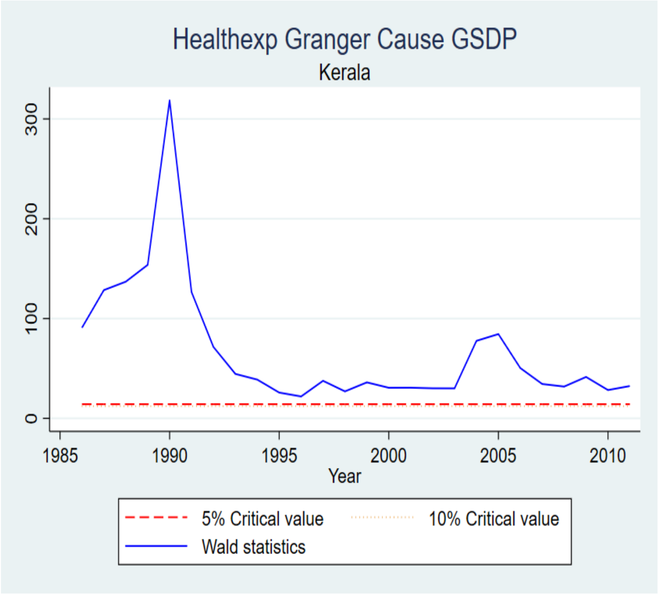

Figure 20

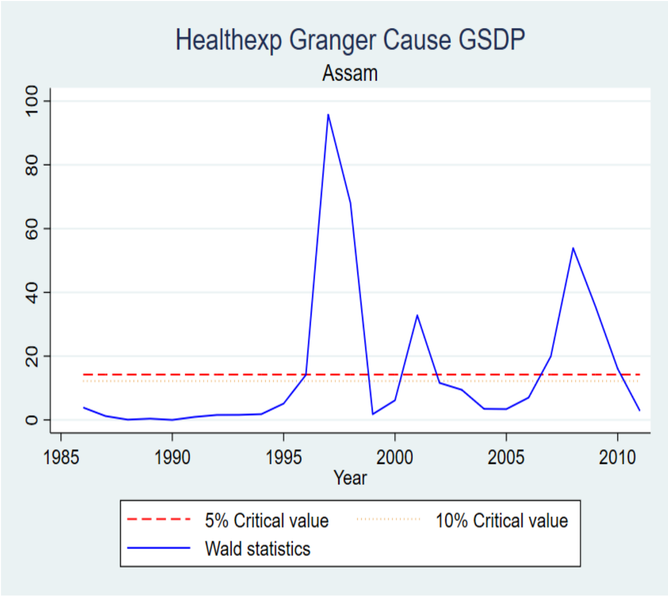

Figure 21

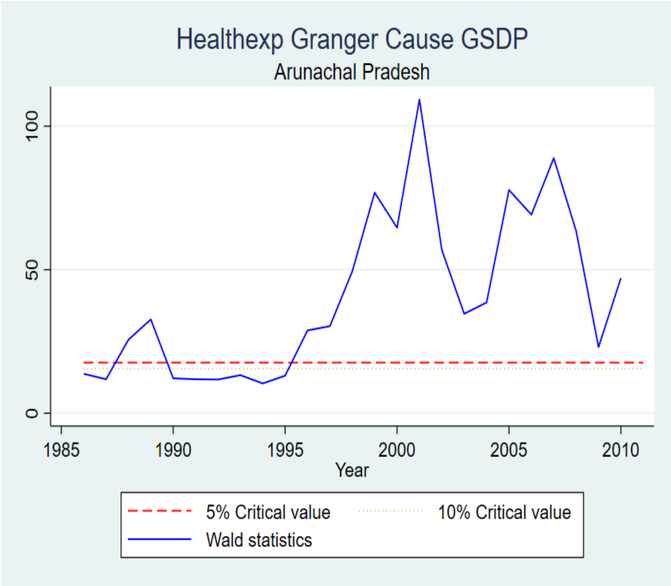

Figure 22

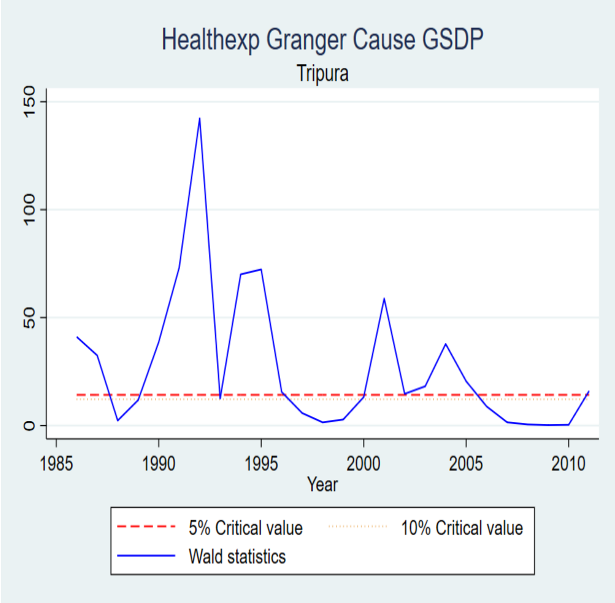

Figure 23

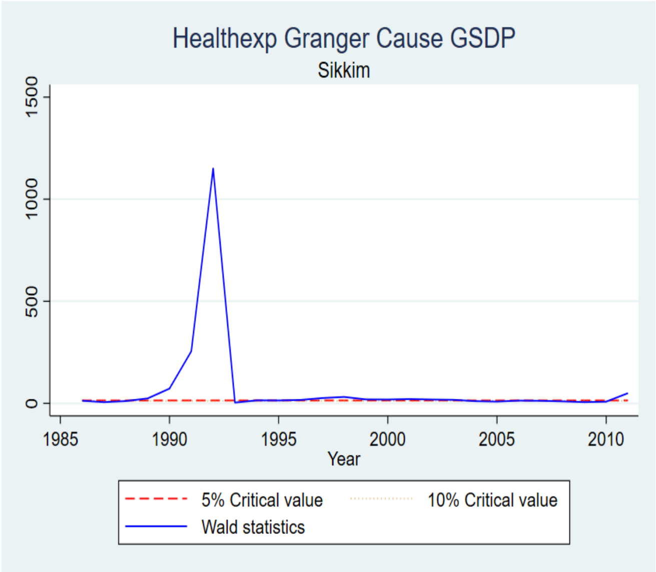

Figure 24

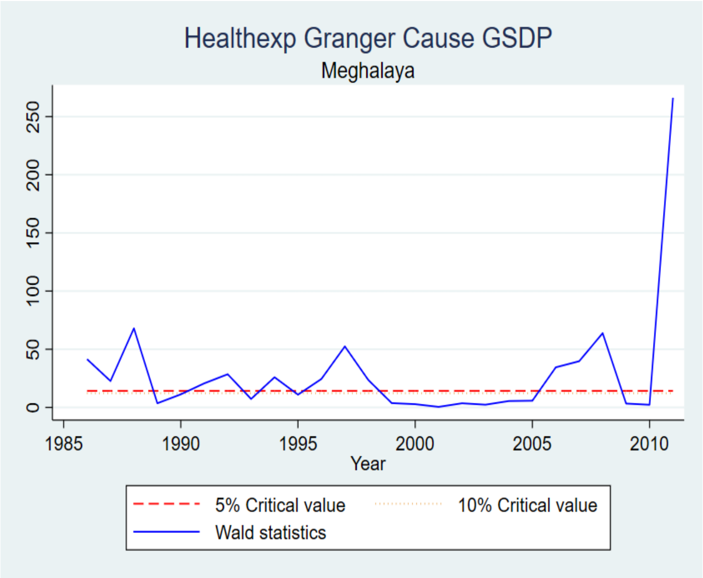

Figure 25

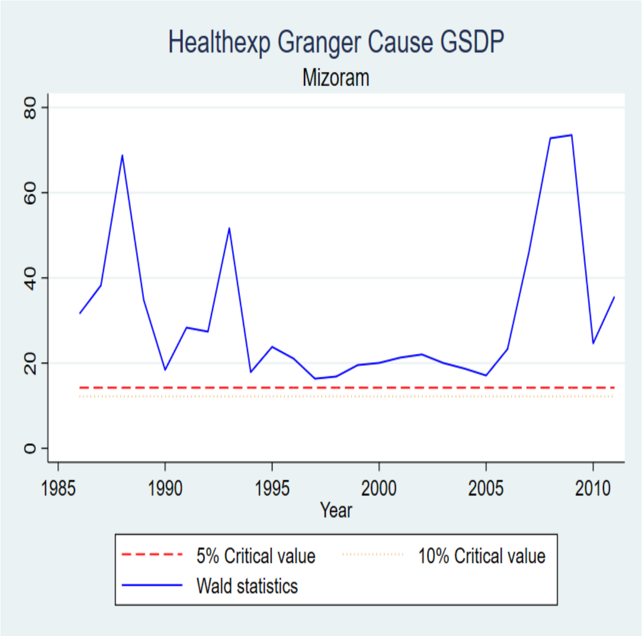

Figure 26

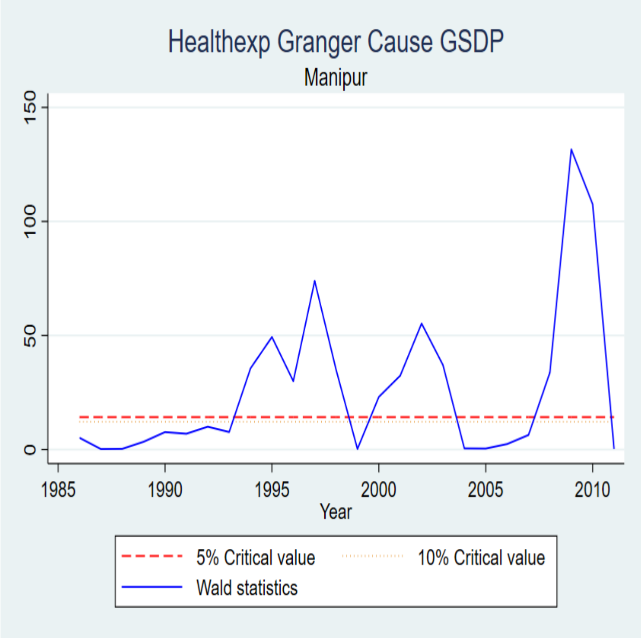

Figure 27

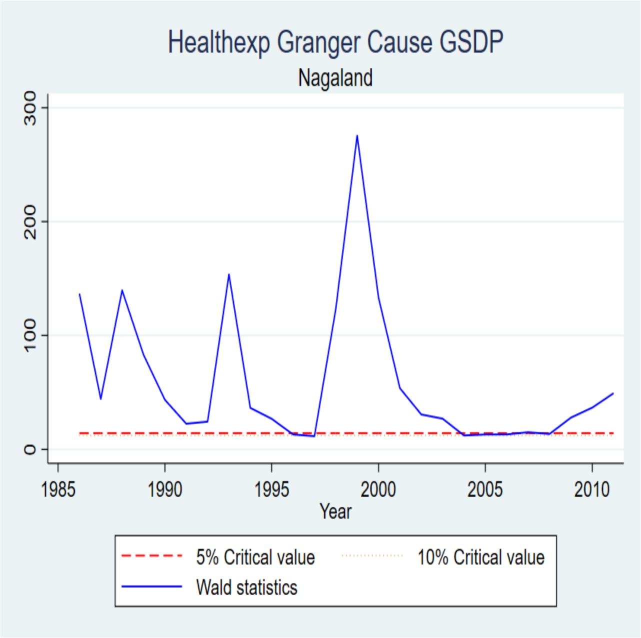

Figure 28

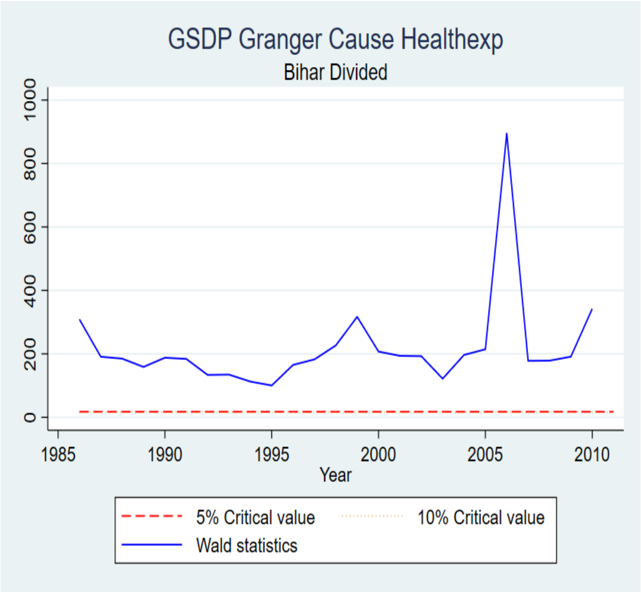

Figure 29

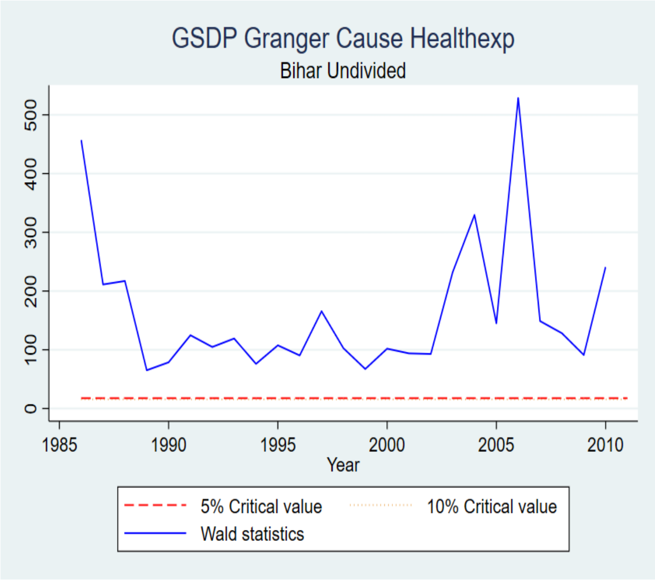

Figure 30

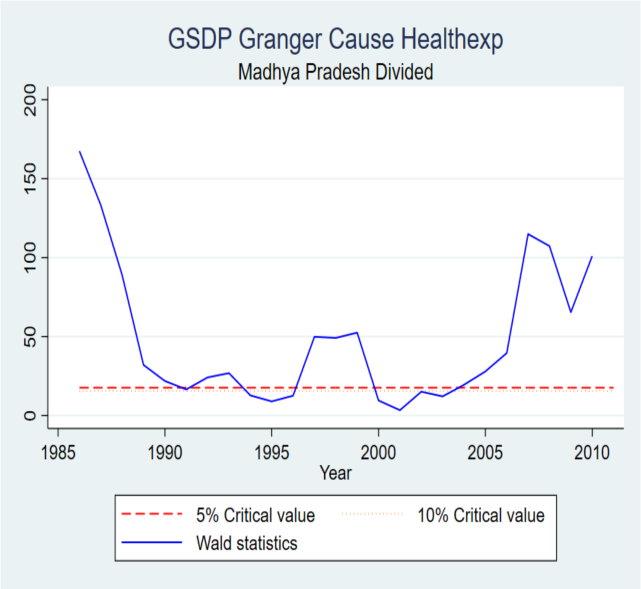

Figure 31

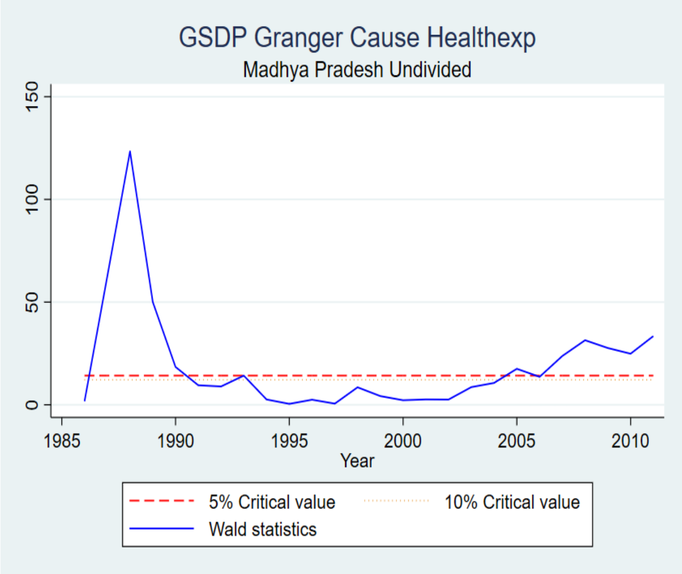

Figure 32

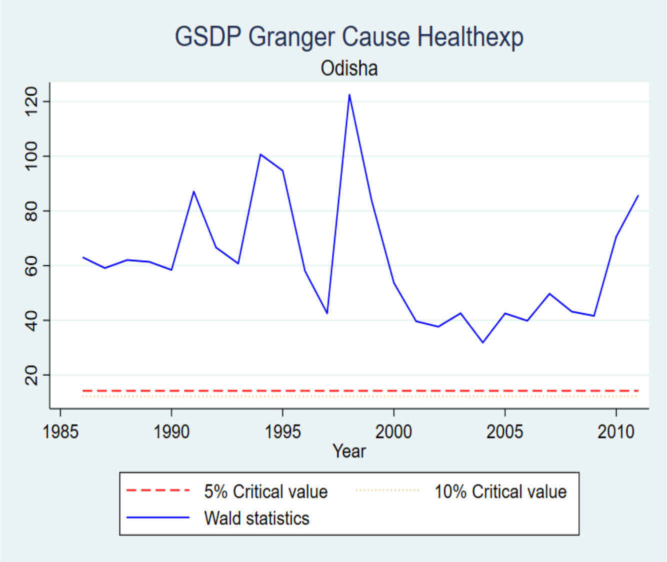

Figure 33

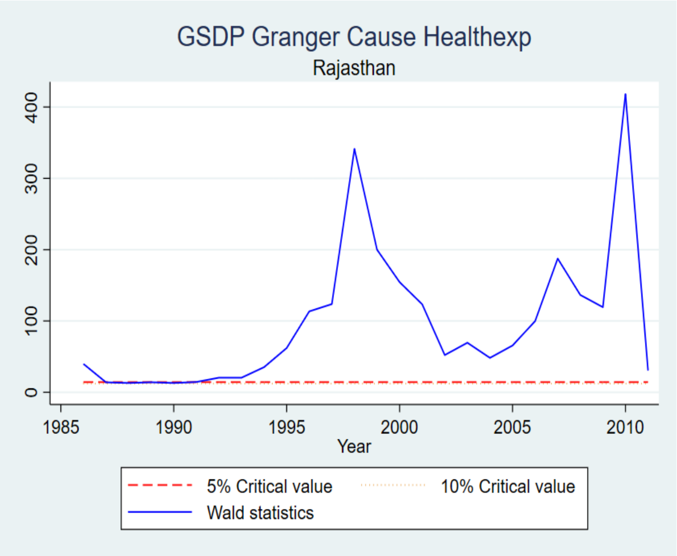

Figure 34

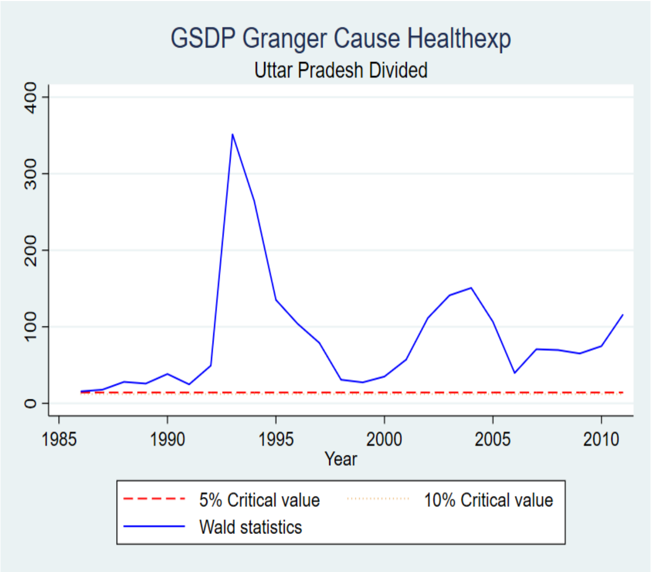

Figure 35

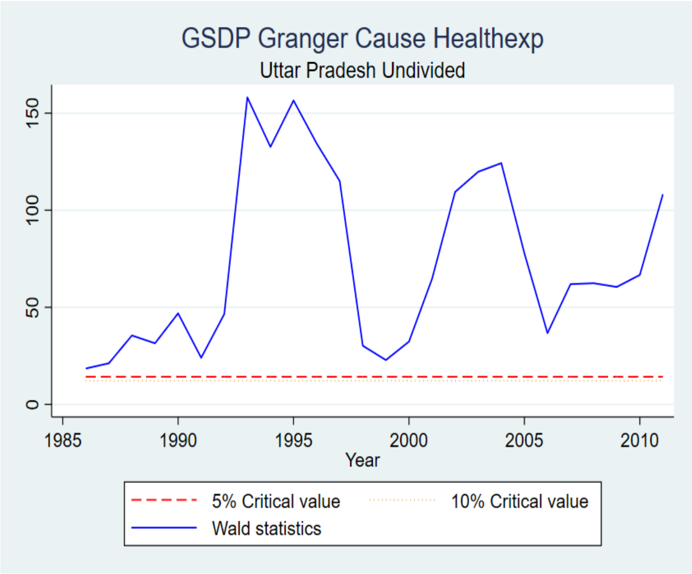

Figure 36

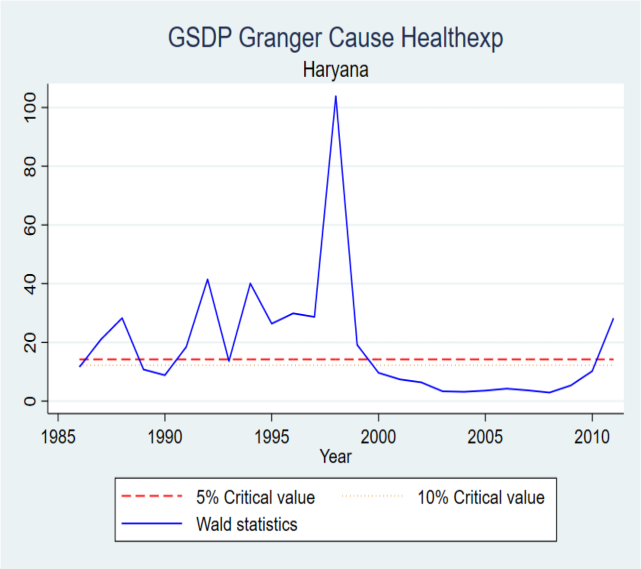

Figure 37

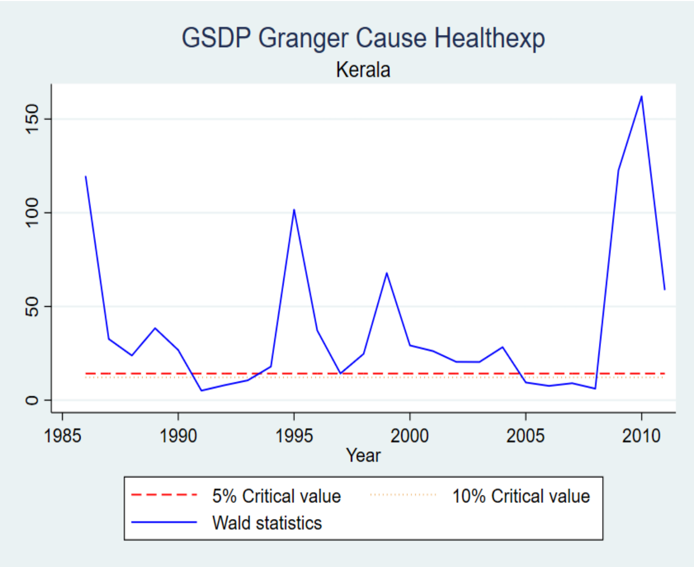

Figure 38

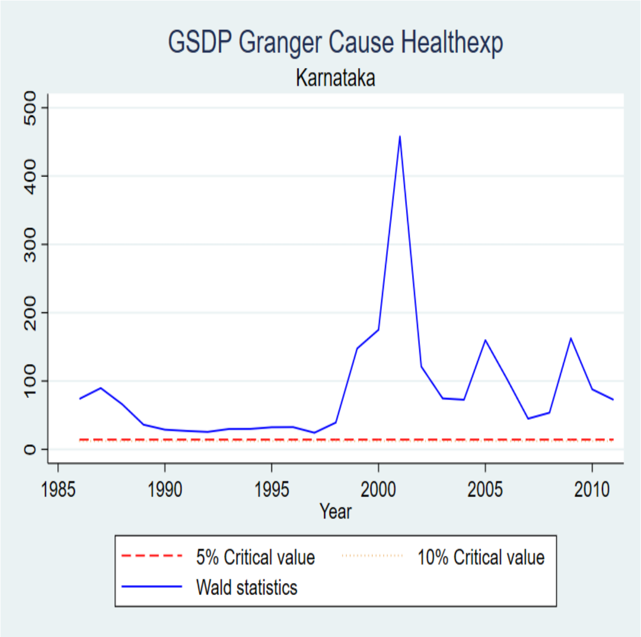

Figure 39

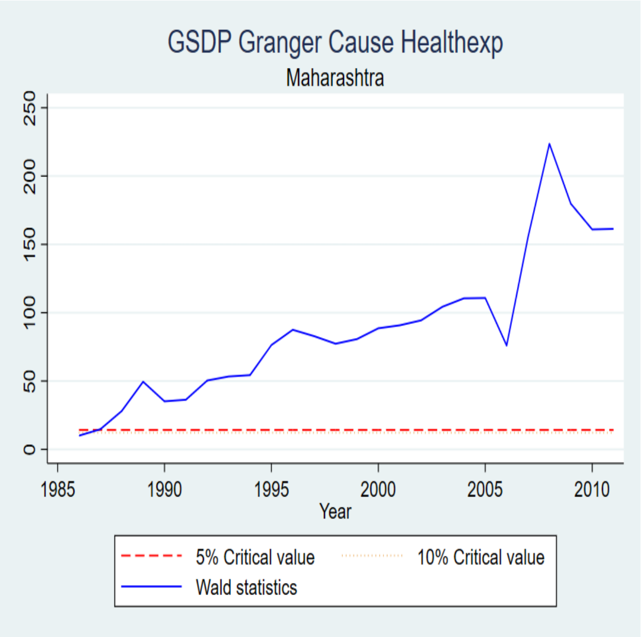

Figure 40

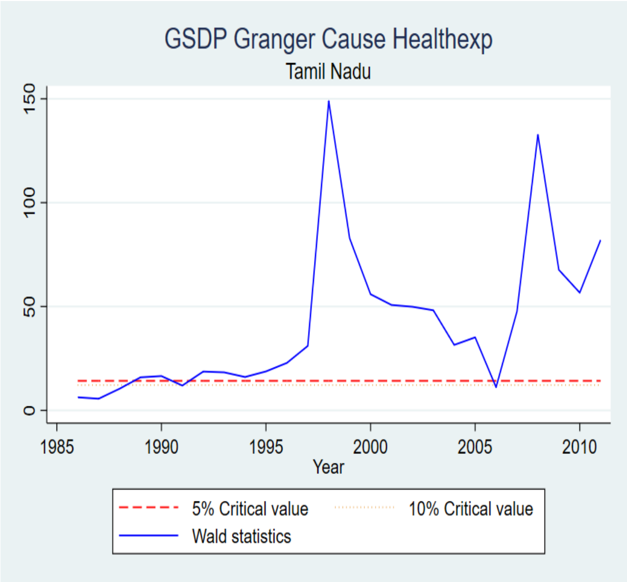

Figure 41

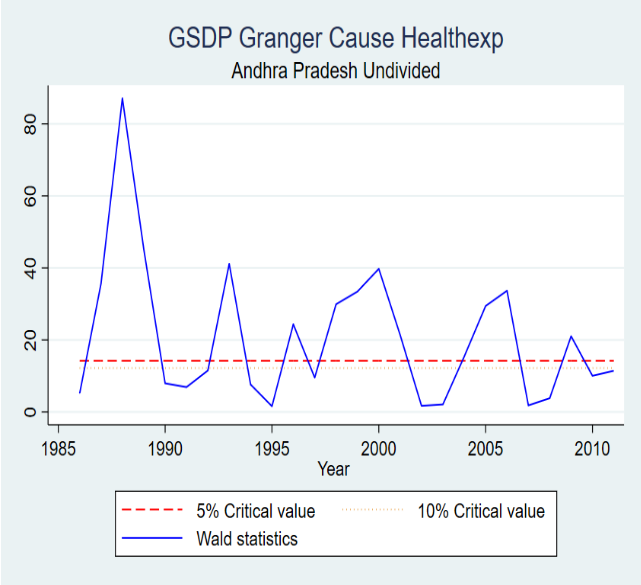

Figure 42

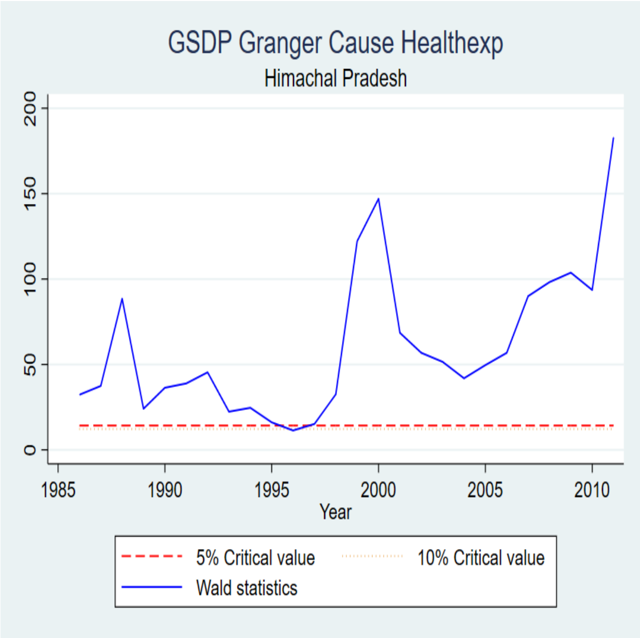

Figure 43

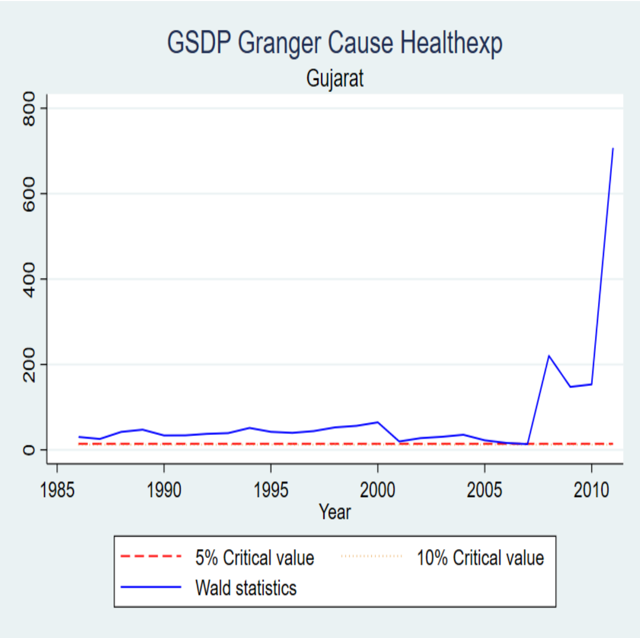

Figure 44

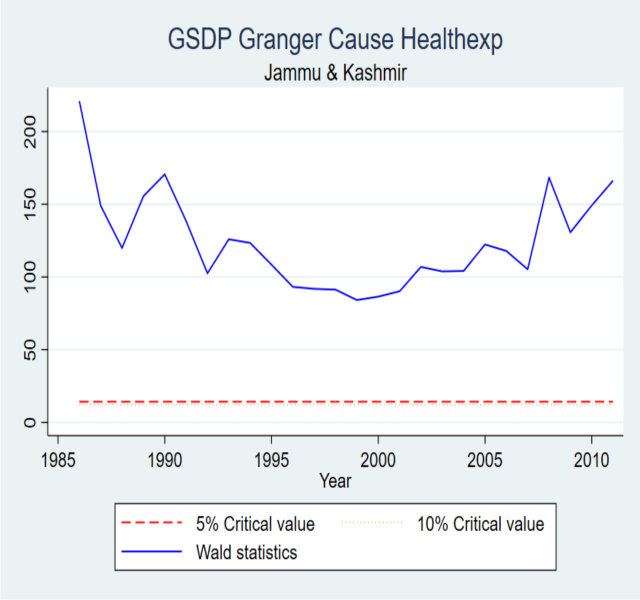

Figure 45

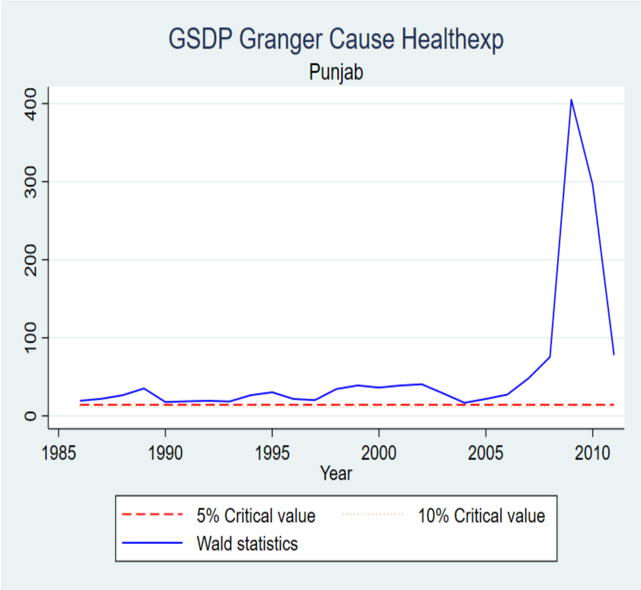

Figure 46

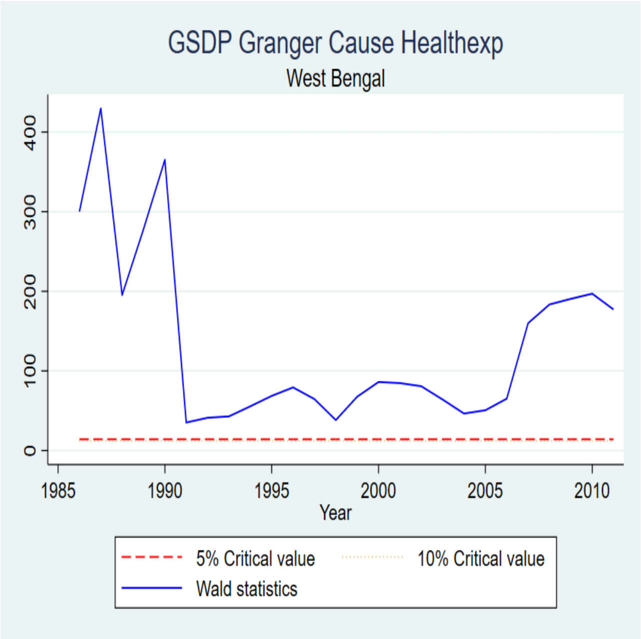

Figure 47

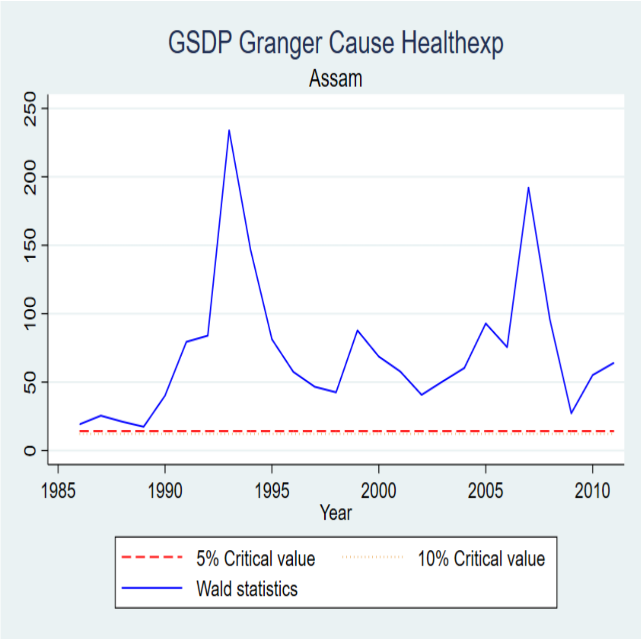

Figure 48

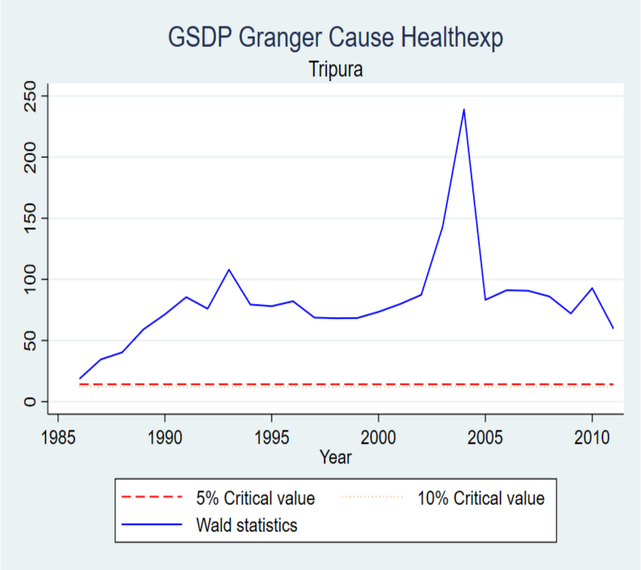

Figure 49

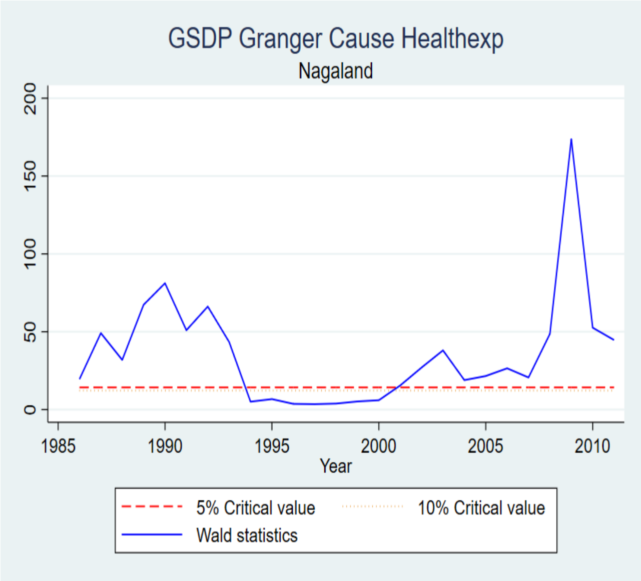

Figure 50

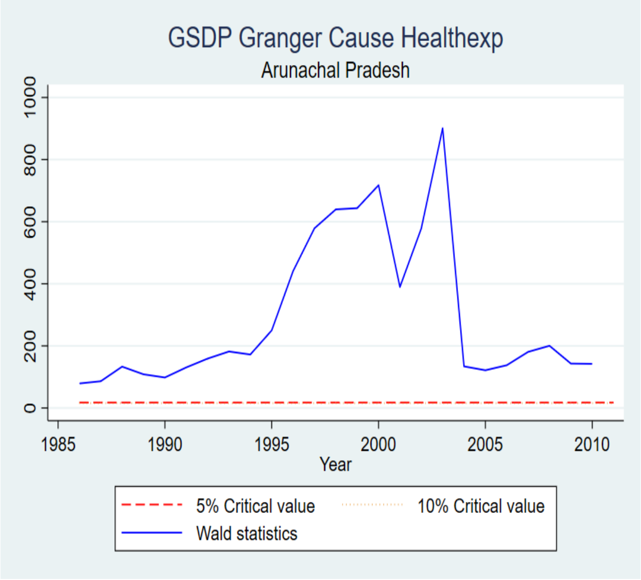

Figure 51

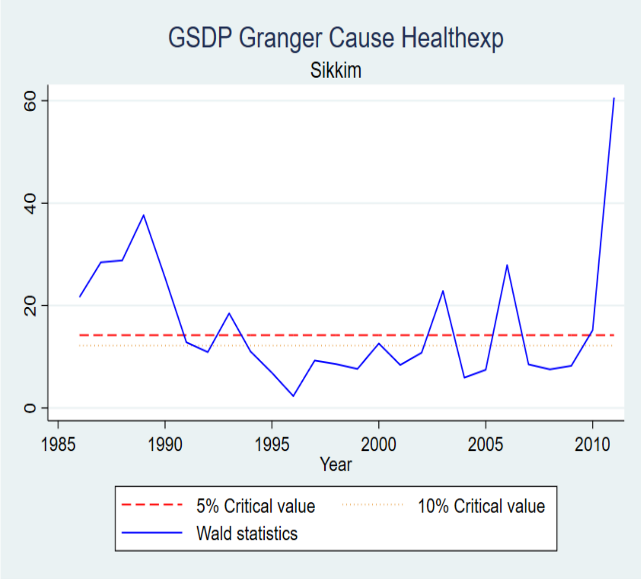

Figure 52

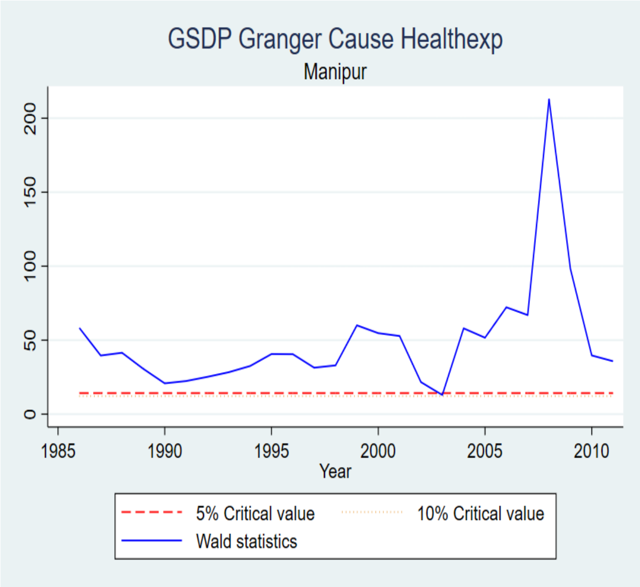

Figure 53

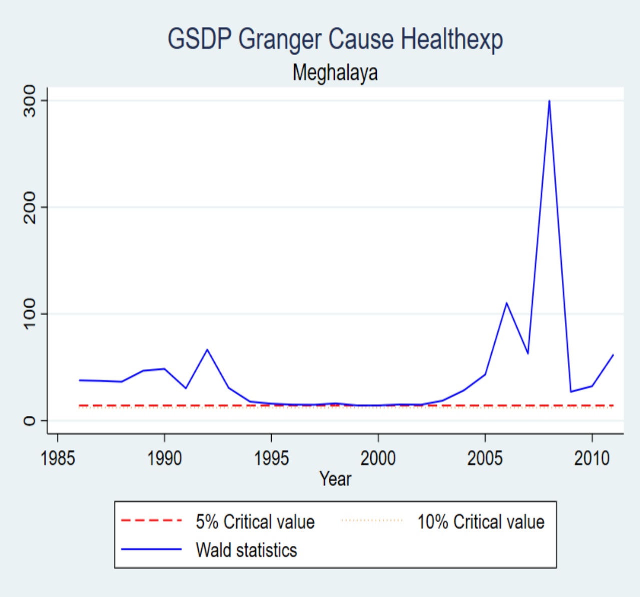

Figure 54

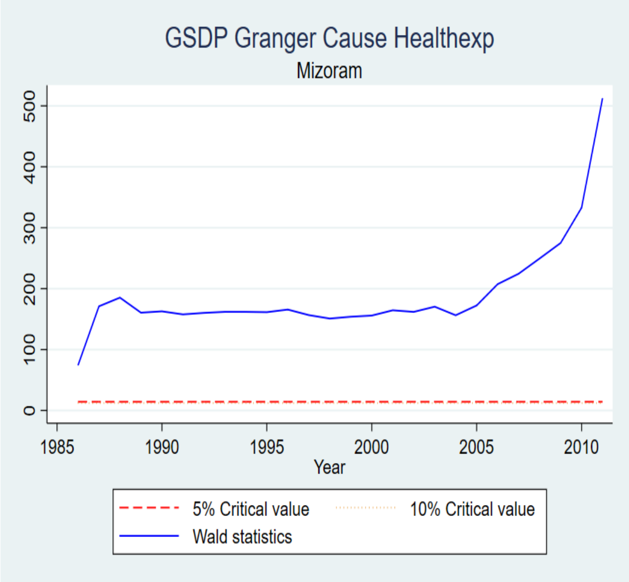

II. Summary Statistics

1. Real Health Expenditure per capita (in INR)

State Group	Observations	Mean	Standard Deviation
a. EAG Undivided	185	274	139
b. Non-EAG Undivided	407	486	288
c. EAG Divided	185	266	134

2. Real GSDP per capita (in INR)

State Group	Observations	Mean	Standard Deviation
a. EAG Undivided	185	27,819	14,157
b. Non-EAG Undivided	407	52,661	31,397
c. EAG Divided	185	26,949	13,869

Notes pertaining to Data

Undivided Bihar is summation of totals of health expenditure and GSDP of Divided Bihar and Jharkhand. The state of Jharkhand was carved out of state of Bihar in 2001.

Undivided Uttar Pradesh is summation of totals of health expenditure and GSDP of Divided Uttar Pradesh and Uttarakhand. The state of Uttarakhand (previously named as Uttaranchal) was carved out of the state of Uttar Pradesh in 2001.

Undivided Madhya Pradesh is summation of totals of health expenditure and GSDP of Divided Madhya Pradesh and Chhattisgarh. The state of Chhattisgarh was carved out of the state of Madhya Pradesh in 2001.

Andhra Pradesh is taken as Undivided AP, because Telangana was carved out of Andhra Pradesh only in 2014.

The population figures are available for district and states only every ten years. The population in the inter-census years is estimated by using the CAGR of the population across ten years. Post 2011, the population projections released by the National Commission on population are used while calculating the state-wise per capita estimates of GSDP and health expenditure.
